# Refined RIP-seq protocol for epitranscriptome analysis with low input materials

**DOI:** 10.1371/journal.pbio.2006092

**Published:** 2018-09-13

**Authors:** Yong Zeng, Shiyan Wang, Shanshan Gao, Fraser Soares, Musadeqque Ahmed, Haiyang Guo, Miranda Wang, Junjie Tony Hua, Jiansheng Guan, Michael F. Moran, Ming Sound Tsao, Housheng Hansen He

**Affiliations:** 1 Princess Margaret Cancer Centre/University Health Network, Toronto, Ontario, Canada; 2 Institute of Digestive Disease and Department of Medicine and Therapeutics, State Key Laboratory of Digestive Disease, Li Ka Shing Institute of Health Sciences, The Chinese University of Hong Kong, Hong Kong; 3 Department of Medical Biophysics, University of Toronto, Toronto, Ontario, Canada; 4 College of Electrical Engineering and Automation, Xiamen University of Technology, Xiamen, China; 5 Program in Cell Biology, Hospital for Sick Children, Toronto, Ontario, Canada; 6 Department of Molecular Genetics, University of Toronto, Toronto, Ontario, Canada; 7 Campbell Family Institute for Cancer Research, Toronto, Ontario, Canada; 8 Department of Laboratory Medicine and Pathobiology, University of Toronto, Toronto, Ontario, Canada; The University of Chicago, United States of America

## Abstract

N6-Methyladenosine (m^6^A) accounts for approximately 0.2% to 0.6% of all adenosine in mammalian mRNA, representing the most abundant internal mRNA modifications. m^6^A RNA immunoprecipitation followed by high-throughput sequencing (MeRIP-seq) is a powerful technique to map the m^6^A location transcriptome-wide. However, this method typically requires 300 μg of total RNA, which limits its application to patient tumors. In this study, we present a refined m^6^A MeRIP-seq protocol and analysis pipeline that can be applied to profile low-input RNA samples from patient tumors. We optimized the key parameters of m^6^A MeRIP-seq, including the starting amount of RNA, RNA fragmentation, antibody selection, MeRIP washing/elution conditions, methods for RNA library construction, and the bioinformatics analysis pipeline. With the optimized immunoprecipitation (IP) conditions and a postamplification rRNA depletion strategy, we were able to profile the m^6^A epitranscriptome using 500 ng of total RNA. We identified approximately 12,000 m^6^A peaks with a high signal-to-noise (S/N) ratio from 2 lung adenocarcinoma (ADC) patient tumors. Through integrative analysis of the transcriptome, m^6^A epitranscriptome, and proteome data in the same patient tumors, we identified dynamics at the m^6^A level that account for the discordance between mRNA and protein levels in these tumors. The refined m^6^A MeRIP-seq method is suitable for m^6^A epitranscriptome profiling in a limited amount of patient tumors, setting the ground for unraveling the dynamics of the m^6^A epitranscriptome and the underlying mechanisms in clinical settings.

## Author summary

N6-Methyladenosine (m^6^A) is one of the most abundant and conserved mRNA modifications. It has been reported to influence multiple steps of RNA life cycle and play an important role in the initiation and progression of human cancers. m^6^A RNA immunoprecipitation followed by high-throughput sequencing (MeRIP-seq) is a powerful technique to map the m^6^A location transcriptome-wide. However, this method typically requires 300 μg of total RNA, which limits its application to patient tumors. In this study, we presented an optimized MeRIP-seq protocol that allows us to profile m^6^A epitranscriptiome using as low as 500 ng of total RNA. By applying our refined protocol to 2 lung cancer patient tumors and integrating with proteomic data, we identified dynamics at the m^6^A level that account for the discordance between mRNA and protein levels in these tumors.

## Introduction

Epitranscriptomics is a functionally relevant change to the transcriptome that depends on biochemical modifications of RNA, and it has emerged as a new layer of post-transcriptional regulation of gene expression [1–3]. To date, more than 150 types of internal RNA modifications have been identified [4]. One particular modification, N6-Methyladenosine (m^6^A), has been characterized as one of the most abundant internal modifications in mammalian mRNAs since its first discovery in the 1970s [1,5–7]. Recent discoveries of the location, function, and molecular mechanism of m^6^A demonstrated that this modification is reversible, undergoes dynamic control, and is regulating multiple steps of the RNA life cycle [1–3], including RNA splicing [8], RNA decay [9], pre-microRNA (pre-miRNA) processing [10], and protein translation [11].

The dynamic m^6^A mRNA modifications have been shown to be closely correlated with differentiation and reprogramming of stem cells, heat shock response, DNA damage response, sex development, and the speed of the circadian clock [12–17]. In addition, recent reports suggest that m^6^A disorders play critical roles in the initiation and progression of human cancers [18,19]. The expression of key m^6^A enzymes, including writers, readers, and erasers, are abnormally altered in many types of tumors. Notably, up-regulation of m^6^A demethylase *ALKBH5* induced by hypoxia causes mRNA demethylation of stem cell marker *NANOG*, leading to increased *NANOG* expression and tumor initiation capacity of breast cancer stem cells [20]. Furthermore, *ALKBH5* also plays an important role in maintaining glioblastoma proliferation and tumorigenesis by enhancing mRNA expression of *FOXM1*, a pivotal transcription factor for glioblastoma stem cell self-renewal [21]. Similarly, oncogenic function of m^6^A demethylase *FTO* and Methyltransferase Like 3 (*METTL3*) has been revealed in acute myeloid leukemia and lung cancer, respectively [22,23].

m^6^A RNA immunoprecipitation followed by next-generation sequencing (m^6^A MeRIP-seq) was developed to identify m^6^A modification transcriptome-wide [24]. More than 10,000 m^6^A sites in the transcripts of approximately 7,000 protein-coding genes and noncoding RNAs have been characterized in human cells [25]. Although several improvements of this technology have been made over the last few years [26] and commercial m^6^A MeRIP kits are available, several dozens to hundreds of micrograms (μg) of total RNA are required [27]. This limits the application of m^6^A epitranscriptome profiling in clinical settings because only single-digit micrograms of total RNA can be obtained from patient samples in most of the cases. Hence, although m^6^A is the most abundant internal modification in mammalian mRNA, the role of m^6^A modification and dynamics in human diseases, especially in cancer, remains largely unknown due to lack of efficient profiling methodologies. Therefore, an m^6^A profiling method that can be applied to low-input RNA samples will provide the basis for a mechanistic understanding of m^6^A dynamics in cancer.

To optimize m^6^A MeRIP-seq for epitranscriptome profiling in patient tumors, we studied the key experimental parameters, namely the starting amount of RNA, RNA fragmentation, m^6^A antibody selection, MeRIP washing/elution conditions, and methods for RNA library construction. Bioinformatics pipelines for peak calling and differential m^6^A analysis were also explored. Using the optimized m^6^A MeRIP-seq protocol, we have successfully identified approximately 12,000 m^6^A peaks with high signal-to-noise (S/N) ratios using 2 μg of total RNA from lung patient tumors. In addition, integrative analysis of transcriptomic, epitranscriptomic, and proteomic profiles in patient tumors identified dysregulated m^6^A sites that account for the discordance between mRNA and protein levels.

## Results

### Optimization of washing/elution conditions

Chemical fragmentation is the first step of m^6^A MeRIP. Due to the feasible conversion of m^1^A to m^6^A at higher temperatures via Dimroth rearrangement, RNA fragmentation was performed in RNA fragmentation buffer at 70 °C instead of 94 °C to minimize m^1^A to m^6^A rearrangement [28,29]. Because we aimed to challenge single-digit microgram total RNA as starting material for m^6^A MeRIP, total RNA rather than poly(A)-enriched mRNA was used for fragmentation. Total RNA was fragmented to a size distribution centered at approximately 200 nt instead of at 100 nt to minimize sample loss (S1 Fig). The quantity of the fragmented RNA centered at approximately 200 nt was 3 times more than that centered at approximately 100 nt.

**Fig 1:**
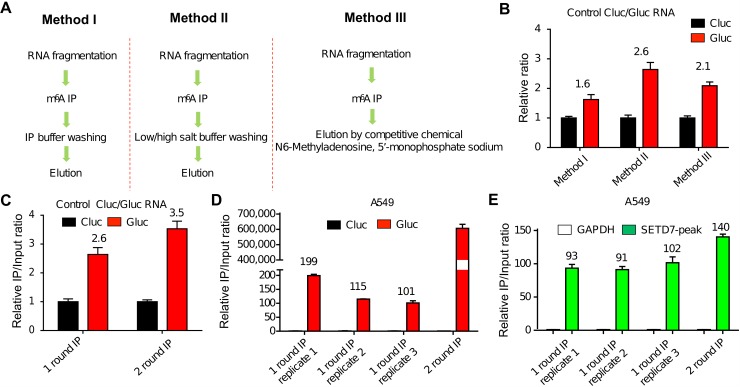
Low/high salt-washing method outperforms competitive elution method. (A) Schematic diagram of 3 strategies of m^6^A MeRIP. (B) S/N ratio of GLuc/CLuc was highest in Method II using low/high salt washing. An RNA mixture containing equal amounts of the m^6^A modified control RNA GLuc, the unmodified control RNA CLuc, and NEB antibody were used for m6A MeRIP. (C) S/N ratio of GLuc/CLuc was further increased in a second round of IP using Method II. S/N ratio of GLuc/CLuc (panel D) and *SETD7*/*GAPDH* (panel E) in 3 replicates of 1 round of IP. Data related to this figure can be found in S1 Data. CLuc, unmodified control RNA; GLuc, m^6^A-modified control RNA; IP, immunoprecipitation; m^6^A, N6-Methyladenosine; m^6^A MeRIP, m^6^A RNA immunoprecipitation followed by high-throughput sequencing; NEB, New England Biolabs; S/N, signal-to-noise.

There are currently two major approaches for m^6^A MeRIP. The first one, designated as Method II (Fig 1A), uses low/high salt washing after immunoprecipitation (IP) incubation [26,30] and subsequently elutes the entire antibody–bead complex. The second approach, designated Method III (Fig 1A), uses IP reaction buffer for washing and m^6^A competition with free m^6^A for elution, which is widely used in the field [24,27,31]. To compare the IP efficiency between these 2 methods, we first performed m^6^A MeRIP using an RNA mixture containing equal amounts of an m^6^A-modified control RNA (GLuc) and an unmodified control RNA (CLuc). The GLuc RNA control was transcribed in vitro in the presence of 20% m^6^ATP and 80% ATP. As a comparison, we added another approach, designated Method I (Fig 1A), which uses IP reaction buffer for washing and elutes the entire antibody–bead complex. Anti-m^6^A antibody from New England Biolabs (NEB) was used in this analysis. Method II, which used low/high salt washing, yielded a higher S/N ratio ([positive m^6^A region IP ÷ Input] ÷ [negative m^6^A region IP ÷ Input]) (Fig 1B). A second round of IP using the eluted RNA from Method II further enhanced the S/N ratio (Fig 1C). Next, we performed m^6^A MeRIP using Method II in RNA samples from the human lung cancer cell line A549. For each RNA pull-down reaction, 0.1 fmol of GLuc and CLuc RNA were spiked into 32 μg of A549 total RNA prior to fragmentation. *SETD7* and *GAPDH* were selected as positive and negative controls, respectively, based on publicly available m^6^A MeRIP-seq data in A549 cells [30]. With 1 round of IP, the S/N ratio of GLuc/CLuc and *SETD7*/*GAPDH* were as high as approximately 100-fold (Fig 1D and 1E). Consistent with the results using pure control RNA GLuc and CLuc, the S/N ratio of GLuc/CLuc and *SETD7*/*GAPDH* were further increased after the second round of IP (Fig 1D and 1E), although the yield was significantly decreased (66%).

### Optimization of m^6^A peak calling and motif analysis pipeline

**Fig 2:**
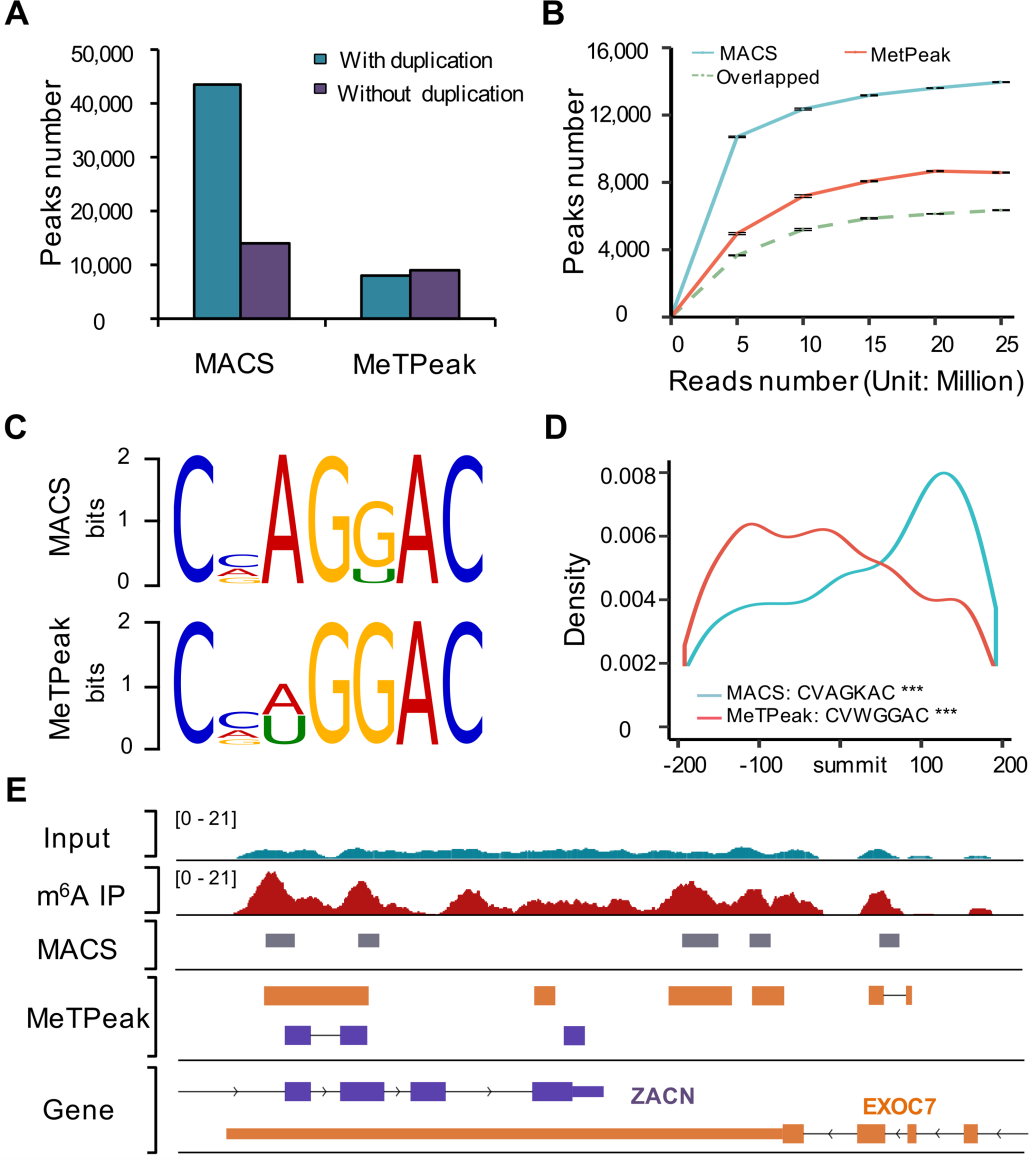
Comparison between MACS- and MeTPeak-based m^6^A detection pipeline with published data from A549. (A) m^6^A peaks detected by MACS and MeTPeak with or without inclusion of duplication reads. (B) m^6^A peaks detected with increasing sequencing depth. The dashed line represents the overlapping peaks called by both MeTpeak and MACS. (C) Top m^6^A motifs detected from top 5,000 m^6^A summit centered at 200-nt peak regions. (D) The location and frequency of the top motif to the summit; ***p < 1 × 10^−4^. (E) Example showing the difference between the results of MACS and MeTPeak. Data related to this figure can be found in S1 Data. IP, immunoprecipitation; m^6^A, N6-Methyladenosine.

Along with the breakthrough of technology for transcriptome-wide m^6^A profiling, 2 analysis approaches have been applied for m^6^A peak calling. The first one employed and tuned the most popular peak calling software, MACS [32], which was originally developed for chromatin immunoprecipitation followed by DNA sequencing (ChIP-seq) analysis, to detect m^6^A sites genome-wide [27]. The second strategy included a series of methods developed specifically for transcriptome-wide m^6^A site detection, such as exomePeak [33], HEPeak [34], and MeTPeak [35]. Because MeTPeak was developed based on exomePeak and HEPeak, we compared the performance of MACS and MeTPeak here using 2 publicly available datasets in A549 (Fig 2) and HepG2 (S2 Fig) cell lines. MACS identified a significantly larger number of m^6^A peaks compared with MeTPeak (Fig 2A and S2A Fig). Most of the peaks detected by MeTPeak overlapped with those detected by MACS (Fig 2B and S2B Fig). Both MACS and MeTPeak are sensitive to sequencing depth, but MeTPeak reaches plateau at a much lower sequencing depth (Fig 2B and S2B Fig). The consensus m^6^A motif “RRACH” (R = G or A; H = A, C, or U) is enriched in m^6^A peaks identified using both approaches; however, the motifs in MeTPeak identified peaks that tend to be closer to the peak summit compared with MACS-identified peaks (Fig 2C and 2D and S2C and S2D Fig). Uniquely identified peaks from MACS tend to be enriched (over 20%) at the transcription start site (TSS) region, with lower enrichment for the m^6^A consensus motif (S2E and S2F Fig). In addition, MeTPeak is more accurate in identifying peaks at junction exons in a strand-specific manner (Fig 2E). With consideration of these results, we employed the MeTPeak-based pipeline for downstream analysis.

### Anti-m^6^A antibody influences the efficiency of m^6^A MeRIP

**Fig 3:**
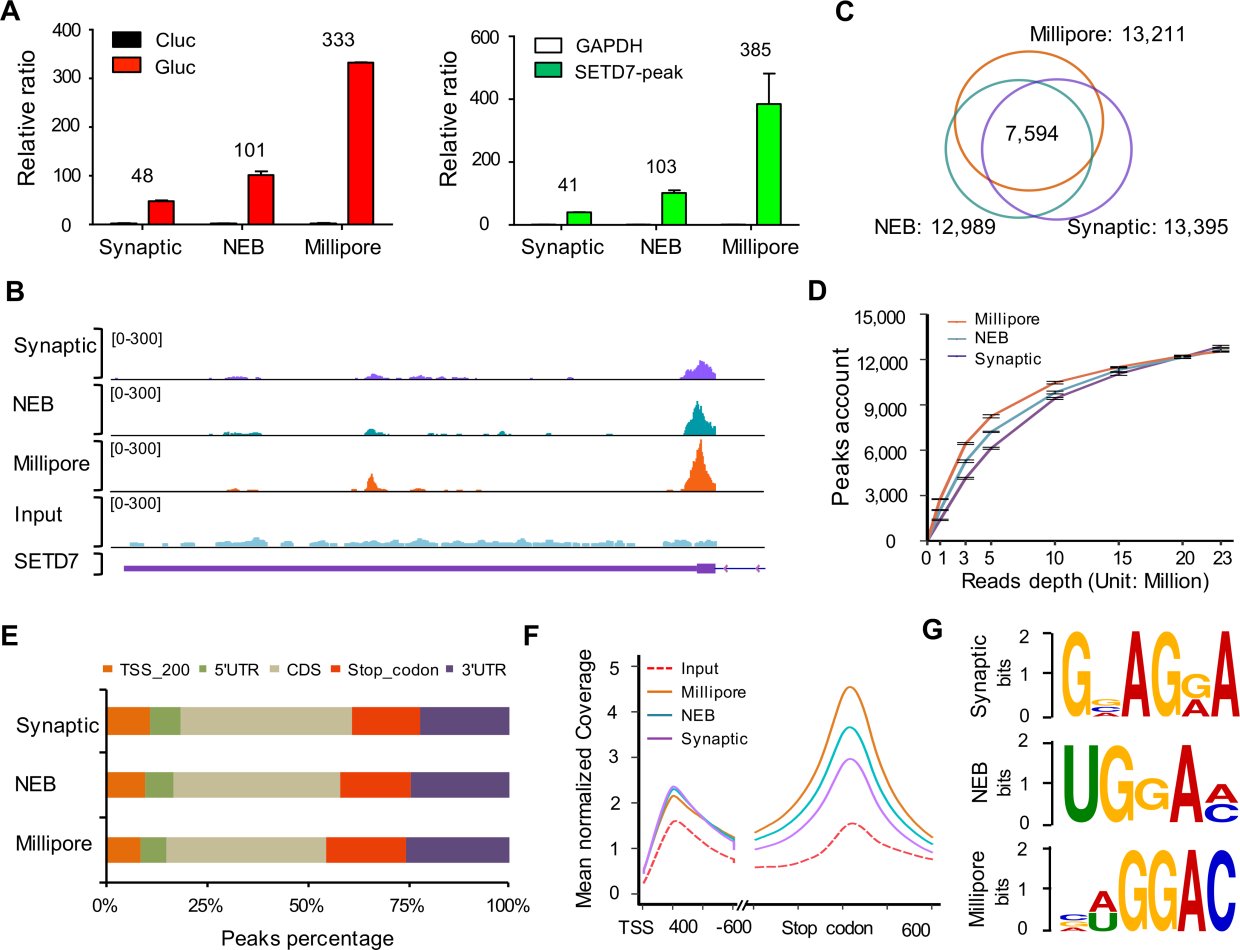
Comparison of 3 different m^6^A antibodies for MeRIP. (A) S/N ratio of GLuc/CLuc and *SETD7*/*GAPDH* with different antibodies. The amount of 32 μg total RNA from human lung cancer cell line A549 with spiked-in control RNA GLuc and CLuc was used for m^6^A MeRIP using Method II. (B) m^6^A peak signals of *SETD7* transcripts in 3 MeRIP-seq libraries. (C) Overlap of m^6^A peaks from the SySy, NEB, and Millipore libraries. (D) Number of m^6^A peaks called by subsampling to different read depths with different antibodies. (E) The percentages of m^6^A peaks in 5 nonoverlapping transcript segments: TSS; 5’UTR; CDS; stop codon; and 3’UTR. (F) Metagene profiles depicting sequence coverage in windows surrounding the TSS and stop codon demonstrated that m^6^A peaks were enriched in the vicinity of the stop codon. (G) Top enriched motifs identified in the SySy, NEB, and Millipore libraries. Data related to this figure can be found in S1 Data. CDS, coding sequence; CLuc, unmodified control RNA; GLuc, m^6^A-modified control RNA; m^6^A, N6-Methyladenosine; m^6^A MeRIP, m^6^A RNA immunoprecipitation followed by high-throughput sequencing; NEB, New England Biolabs; S/N, signal-to-noise; SySy, Synaptic Systems; TSS, transcription start site; UTR, untranslated region.

Having optimized the m^6^A MeRIP protocol and computational analysis pipeline, we next sought to compare the fidelity of anti-m^6^A antibodies through MeRIP-seq analysis. Three anti-m^6^A antibodies from Synaptic Systems (SySy), NEB, and Millipore were chosen for this analysis. From A549 cells, 32 μg of total RNA were used in each RNA pull-down reaction, and control RNA GLuc and CLuc were spiked in prior to RNA fragmentation as described above. The total MeRIP yield with SySy antibody was twice as much as that of NEB or Millipore antibodies. The S/N ratio as measured by GLuc/CLuc and *SETD7*/*GAPDH* was high for all 3 antibodies, with both NEB and Millipore antibody having a ratio more than 100 (Fig 3A).

After comparing a few commercially available RNA sequencing (RNA-seq) library preparation kits, we applied the SMARTer Stranded Total RNA-Seq Kit (Pico Input Mammalian) from Takara/Clontech for library construction in our study. This kit applies a strategy to remove ribosomal cDNA using probes specific to mammalian rRNA post reverse transcription and amplification. It avoids depleting rRNA or enriching for poly(A)^+^ RNA prior to RNA fragmentation, which greatly reduces RNA loss for low-input RNA samples. Using this kit, we successfully constructed sequencing libraries for all the IP and input RNA samples generated with the 3 antibodies (S1 Table). In agreement with the quantitative reverse transcription PCR (RT-qPCR) result, a similar trend of m^6^A peak S/N ratio among these 3 antibodies was observed for *SETD7* transcript (Fig 3B). Around 13,000 m^6^A peaks were detected in each library (Fig 3C), with 3 peaks per gene on average (S3 Table). Approximately 60% m^6^A peaks overlapped among these 3 libraries (Fig 3C). The peak numbers converge when a plateau is reached at a sequencing depth of 20 M reads for all 3 antibodies (Fig 3D). The distribution of m^6^A peaks along transcripts has been well characterized to be enriched in the vicinity of the stop codon [24]. We observed a similar m^6^A percentage distributed in 5 nonoverlapping genome features for all 3 antibodies (Fig 3E; *p* = 0.997), and consistent m^6^A signal enriched near the stop codon (Fig 3F). In addition, m^6^A peaks in both Millipore and NEB libraries have the enrichment for core m^6^A motif “GGAC” (Fig 3G). Finally, our 32-μg libraries are also comparable to the aforementioned publicly available A549 dataset starting with 300 μg total RNA (S3A Fig, S1, S3 and S4 Tables).

### m^6^A MeRIP-seq with low-input RNA from A459 cells

**Fig 4:**
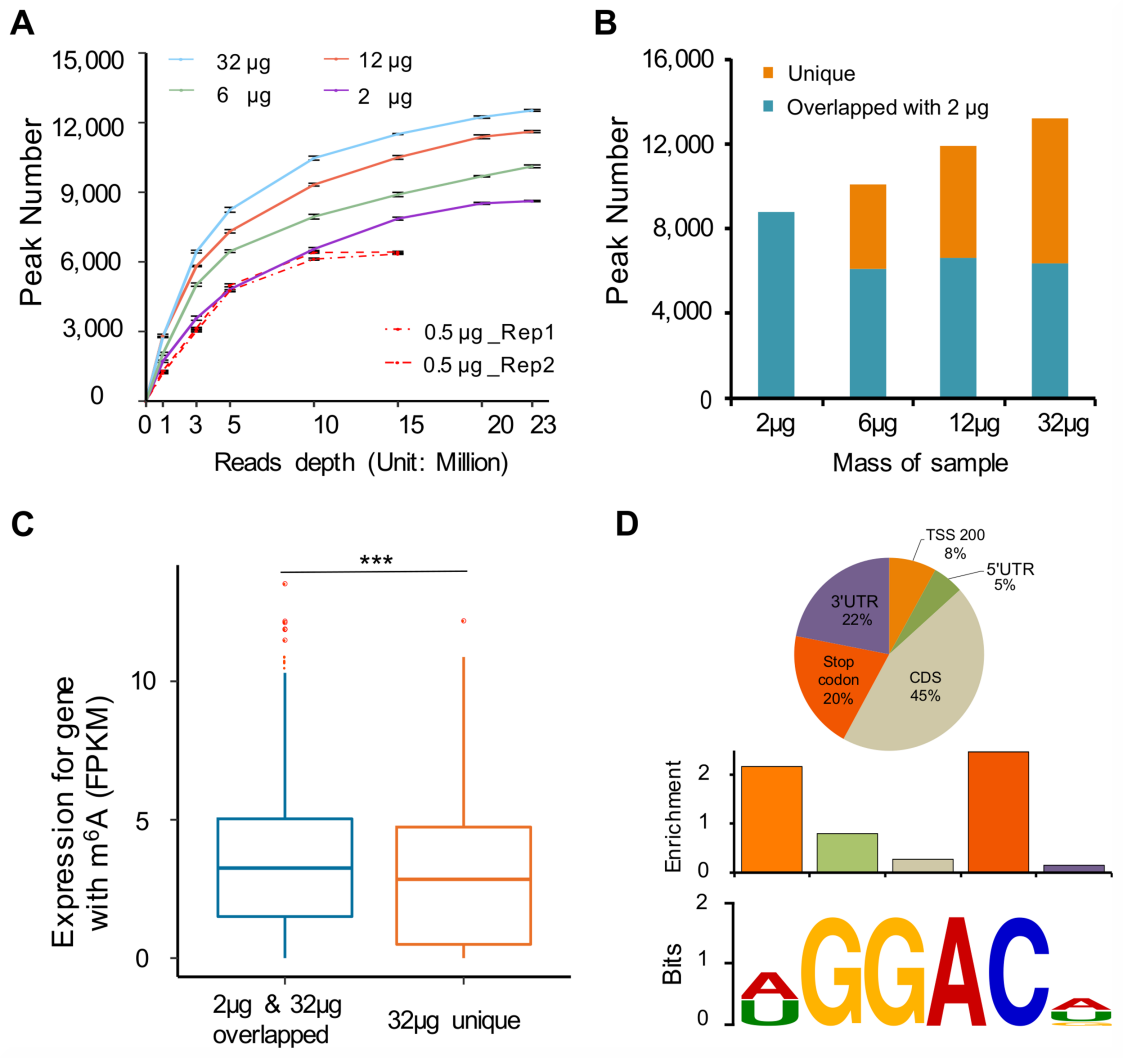
Optimized m^6^A MeRIP-seq protocol worked well starting with 2 μg total RNA. (A) MeRIP efficiency decreases with the reduction of starting RNA amount. (B) At the same sequencing depth, the total number of m^6^A peaks identified increased with the increase of starting RNA amount. The “unique” and “overlapped with 2 μg” peaks indicate the peak number compared to 2 μg. (C) The average RNA expression level of the transcripts with unique m^6^A peaks identified in the 32-μg library was significantly lower than that of overlapping m^6^A peaks; ***p < 1 × 10^−4^. (D) Top: pie chart represents the proportion of m^6^A peaks in each of the 5 nonoverlapping transcript segments in the 2-μg library. Middle: relative enrichment of m^6^A peaks across the 5 nonoverlapping transcript segments in the 2 μg library. Bottom: top enriched motifs in the 2 μg library. Data related to this figure can be found in S1 Data. CDS, coding sequence; m^6^A, N6-Methyladenosine; m^6^A MeRIP, m^6^A RNA immunoprecipitation followed by high-throughput sequencing; TSS, transcription start site; UTR, untranslated region.

We next examined the m^6^A MeRIP efficiency by testing different amounts of RNA (32 μg, 12 μg, 6 μg, 2 μg, 1 μg, and 0.5 μg) as starting material using the Millipore antibody. The S/N ratio of *SETD7*/*GAPDH* gradually decreased with the reduction of starting amount of total RNA (S4A Fig). The reproducibility was high as exemplified with 0.5 μg total RNA samples (S4A and S4B Fig and S2 Table; r = 0.9). Increasing the starting RNA amount increased the number of m^6^A peaks detected, and the peak number reached plateau at around 20 M reads, except for 0.5 μg, which reached plateau at around 10 M reads (Fig 4A). When we compared 32 μg, 12 μg, and 6 μg to the 2-μg library, we observed that increasing the starting RNA amount led to a greater number of unique m^6^A peaks (Fig 4B). Furthermore, the average expression level of the genes (*N* = 1,579) with overlapping m^6^A peaks was significantly higher compared with genes (*N* = 2,168) with unique m^6^A peaks identified in the 32-μg library (Fig 4C), indicating that the m^6^A peaks in the genes with relatively less abundance could be characterized in m^6^A MeRIP-seq with more starting RNA amount. The enrichment of m^6^A peaks near the stop codon and the m^6^A consensus core motif observed in the 2-μg library are comparable to the other libraries with a greater starting RNA amount (Fig 4D and S3B–S3E Fig). Moreover, our 2-μg library is also comparable to the control dataset using 300 μg RNA (S3F and S3G Fig, S1, S3 and S4 Tables).

We also examined the ratio between antibody and RNA using 5 μg, 1 μg, and 0.2 μg Millipore antibody for 2 μg total RNA. Lowering the quantity of the antibody resulted in increased S/N ratio but with the cost of decreased RNA yield (S4C Fig).

### m^6^A epitranscriptome profiling in lung adenocarcinoma tumors

**Fig 5:**
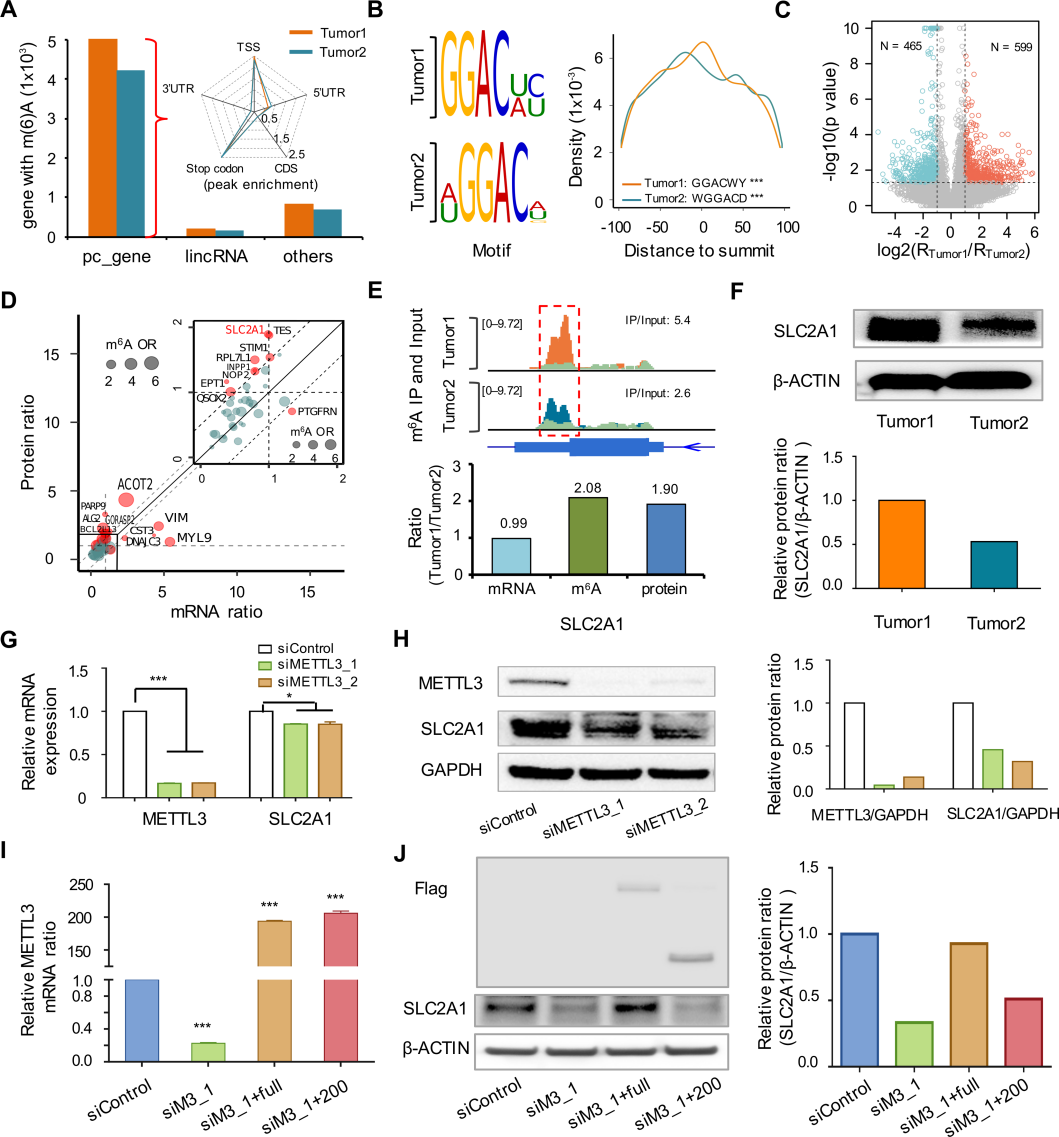
m^6^A dynamics in ADC tumors. (A) Transcriptome-wide distribution of m^6^A sites. (B) Top motif and their distance to summit of m^6^A peaks; ***p < 1 × 10^−4^. (C) Volcano plot for peaks with differential m^6^A intensity between tumor1 and tumor2. R_Tumor1_ and R_Tumor2_ stand for the ratio of IP over Input for sample tumor1 and tumor2, respectively. Peaks with *p* < 1 × 10^−10^ were reassigned to be *p* = 1 × 10^−10^. (D) Correlation between the ratios (tumor1/tumor2) at RNA and protein levels for genes with differential m^6^A peaks. Red dots represent genes with discordance ratio at mRNA and protein levels, and dot size represents m^6^A odds ratio between tumor1 and tumor2. (E) Top: the IP (orange for tumor1 and cyan for tumor2) and Input (light green) signal at m^6^A peak (marked by red box) near *SLC2A1* stop codon in the 2 tumors. Bottom: ratio between tumor1 and tumor2 at mRNA and protein levels for *SLC2A1*. (F) Western blotting analysis of tumor1 and tumor2 samples. (G) Real-time PCR analysis of *METTL3* and *SLC2A1* mRNA expression levels in A549 upon silencing of *METTL3* using 2 different siRNAs. *p < 0.05; ***p < 1 × 10^−4^. (H) Western blotting analysis of *METTL3* and *SLC2A1* protein levels in the A549 upon *METTL3* knockdown. (I) Real-time PCR analysis of *METTL3* mRNA in rescue assays. *METTL3* primer recognized both full-length and 1–200AA *METTL3* mutant. *METTL3* knockdown cells were transfected with either full-length *METTL3* (siM3_1+full) or 1–200AA *METTL3* mutant overexpressing plasmid (siM3_1+200). siM3_1 is the abbreviation for siMETTL3_1; ***p < 1 × 10^−4^. (J) Western blotting analysis of *SLC2A1* in rescue assays. Data related to this figure can be found in S1 Data. ADC, adenocarcinoma; CDS, coding sequence; IP, immunoprecipitation; lincRNA, long intergenic noncoding RNA; m^6^A, N6-Methyladenosine; METTL3, Methyltransferase Like 3; siRNA, small interfering RNA; *SLC2A1*, Solute Carrier family 2, Facilitated Glucose Transporter member 1; TSS, transcription start site; UTR, untranslated region.

Having demonstrated the fidelity of the refined m^6^A MeRIP-seq protocol for low-input RNA from a human cell line, we then applied the protocol to profile the m^6^A epitranscriptome in 2 adenocarcinoma (ADC) patient tumors (tumor1 and tumor2). To control for systematic variations of the MeRIP experiment, spike-in RNA was introduced [36,37]. Because abundant m^6^A RNA modification was detected in bacteria K-12 [38], 9 ng of K-12 total RNA was added to 2 μg of patient tumor RNA prior to RNA fragmentation (S5 Fig). We detected approximately 12,000 m^6^A peaks in each tumor, and approximately 60% of them were observed in both tumors (S6A Fig). The majority of the m^6^A peaks were found at the stop codon and TSS of protein coding genes (Fig 5A), with the consensus m^6^A motif enriched close to the summit of the peaks in each tumor (Fig 5B). To further identify differential m^6^A peaks, we merged the peaks in both tumors and calculated tag counts in IP and input samples for each individual peak. A Fisher exact test of the normalized tag counts identified 599 peaks (554 genes) with higher m^6^A intensity in tumor1 and 465 peaks (417 genes) with higher m^6^A intensity in tumor2 (Fig 5C and S6B–S6D Fig). To further explore the effect of differential m^6^A on protein levels, we performed mass spectrometry (MS) analysis of both tumors. Out of the 953 genes with differential m^6^A peaks, 209 can be detected by MS. We further narrowed them down to 45 genes with relatively high abundance at both mRNA (Reads Per Kilobase of transcript per Million mapped reads [RPKM] > 1) and protein (Tumor_light_/Stable Isotope Labeling by Amino acids in Cell culture [SILAC]_heavy_ > 0.5) levels, and 18 of these 45 genes have discordant fold changes between the 2 tumors at RNA and protein levels (Fig 5D). As exemplified in the case of Solute Carrier family 2, Facilitated Glucose Transporter member 1 (*SLC2A1*), while the mRNA levels were similar between the 2 tumors, the protein level was 1.9-fold higher in tumor1 compared with tumor2 (Fig 5E). The protein expression in tumor1 and tumor2 was further verified by western blotting analysis using patient-derived xenograft (PDX) tissues (Fig 5F). The m^6^A level near the stop codon of *SLC2A1* was about 2-fold higher in tumor1 (Fig 5E). We therefore hypothesized that the discordance between mRNA and protein levels might be due to the dynamics at the m^6^A level. To test this hypothesis, we knocked down *METTL3* using 2 different small interfering RNAs (siRNAs) in the A549 cell line. The knockdown efficiency was confirmed by both RT-qPCR and western blot (Fig 5G and 5H). The protein level of *SLC2A1* was dramatically down-regulated with a slight difference at the mRNA level upon knockdown of *METTL3* (Fig 5H). The noncatalytic domain of *METTL3* (1–200 amino acids [AAs]) has been reported to be able to drive Epidermal Growth Factor Receptor (*EGFR*) protein translation [22]. We therefore investigated whether enhanced translation efficiency of *SLC2A1* by *METTL3* was dependent of its catalytic activity. Full-length, but not the catalytic-domain–truncated, *METTL3* (1–200AA) rescued *SLC2A1* protein expression in the endogenous *METTL3* knockdown cells (Fig 5I and 5J), suggesting that m^6^A methylation promotes the translation efficiency of *SLC2A1* in ADC.

## Discussion

In this study, we optimized the performance of m^6^A MeRIP-seq for epitranscriptome analysis in patient tumors. Applying the optimized protocol (S1 Text), we successfully profiled the m^6^A epitranscriptiome using 2 μg of total RNA from 2 lung cancer patient tumors. m^6^A modification is highly dynamic and cell-context dependent [7]. Of the rhythmic genes controlled by the circadian clock in the liver, only one-fifth are driven by de novo transcription and most are controlled by m^6^A-dependent RNA processing [14]. Site-specific cleavage and radioactive labeling followed by ligation-assisted extraction and thin-layer chromatography (SCARLET), a method that accurately determines m^6^A status at a specific site, has shown that the m^6^A modification levels at specific sites in several mRNAs and long noncoding RNAs (lncRNAs) varied greatly in different cell lines [39]. In this regard, we analyzed the dynamics of m^6^A profiles in the 2 lung ADC patient tumors and identified a few hundred differential m^6^A peaks between the 2 tumors.

The correlation between mRNA and protein expression is poor for many genes [40]. Only approximately 40% of the variation at protein level can be explained by mRNA abundance [40]. The discordance between mRNA and protein levels may at least be partially due to post-transcriptional RNA modifications. m^6^A plays a critical role in cap-independent and -dependent translation dynamics [7]. Specifically, m^6^A readers *YTHDF1* and *YTHDF3* promote the protein translation of targeted mRNAs by interacting with the translation machinery [11,41]. The depletion of m^6^A methyltransferase *METTL3* reduces the translation efficiency of genes with m^6^A modification, such as *c-MYC*, *BCL2* and *PTEN*, and promotes cell differentiation in acute myeloid leukemia [42]. However, *METTL3* can also interact with components of the *eIF3* translation initiation complex and facilitates the translation of oncogenes such as *EGFR* and *TAZ* independent of m^6^A reader proteins, which contributes to tumorigenesis [22]. On the contrary, a recent report examining 135 human promoters observed that m^6^A modification deposition was cotranscriptional and negatively correlated with transcription elongation [43]. Reduced transcription rates led to elevated m^6^A deposition, which resulted in decreased translation [43]. Therefore, whether m^6^A promotes or inhibits translation efficiency is dependent on the downstream m^6^A readers. Integrative analysis of the m^6^A profiling data, RNA-seq data, and MS data from the 2 ADC identified 18 genes with differential m^6^A levels and discordant changes at mRNA and protein levels. In tumor1, the protein levels were significantly up-regulated compared with the mRNA levels for several genes such as *ACOT2*, *PARP9*, *ALG2*, and *SLC2A1*. On the other hand, our analysis also found that the protein levels of a few genes, such as *MYL9*, *VIM*, and *CST3*, were significantly down-regulated relative to the mRNA levels in tumor2. To what extent the m^6^A modification can explain the discordance between the mRNA and protein levels warrants further investigation.

In summary, our refined m^6^A MeRIP-seq protocol and analysis pipeline will expedite the epitranscriptome profiling in patient tumors, which will not only set the ground for understanding m^6^A dynamics in cancer development and progression but also provide opportunities for development of m^6^A-related new biomarkers and therapeutic targets to improve cancer management. It is worth noting that our protocol may be expanded to profile other RNA modifications, such as m^5^C and m^1^A, when good antibodies become available. Additional optimizations, such as integration of molecular barcodes, may further improve the performance of the protocol.

## Materials and methods

### Ethics statement

The protocol (13-6068TE) for collection of tumor samples from surgically resected NSCLC was approved by The University Health Network Human Research Ethics Committees. Patient tumor samples were obtained by informed consent.

### Antibodies and plasmids

We used the following antibodies to m^6^A: rabbit polyclonal anti-m^6^A (202 003, SySy, Germany; ABE572, Millipore, Germany) and rabbit monoclonal anti-m^6^A supplied in EpiMark N6-Methyladenosine Enrichment Kit (E1610S, NEB, Ipswich, MA). pCMV-Flag-MS2-METTL3 full-length and pCMV-Flag-MS2-METTL3 1–200AA plasmids were generously provided by Professor Richard I. Gregory (Harvard University).

### Cell lines and tumor samples

Lung cancer cell line A549 was obtained from the American Type Culture Collection (ATCC; Manassas, VA). Cell line A549 was maintained according to protocols from ATCC. Surgically resected samples from primary lung tumor were obtained from lung cancer patients at the time of operation before any therapeutic intervention, as described previously [44]. Two patients with confirmed lung cancer were examined for m^6^A MeRIP. The study protocol was approved by the Clinical Research Ethics Committee of UHN.

### RNA extraction and DNAse treatment

Total RNA from cells in culture was extracted using Trizol reagent (15596018; Thermo Fisher Scientific, Waltham, MA). Due to the presence of m^6^A in DNA [45], total RNA was treated with DNase I (04 716 728 001; Roche Diagnostics, Indianapolis, IN) for 20 minutes at 37 °C to remove DNA contamination. The RNA was precipitated using glycogen (25 μg/mL final) (5 mg/mL; AM9510; Thermo Fisher Scientific) and isopropanol at −30 °C for 2 hours. The precipitated RNA was then washed with 70% ethanol. The final pellet was resuspended in ultrapure H_2_O. The concentration of total RNA was measured by Qubit RNA HS Assay Kit (Q32855; Thermo Fisher Scientific).

### RNA fragmentation

RNA fragmentation is based on the previously described m^6^A-seq protocol [27] with a few modifications: the total volume of approximately 3 to 5 μg total RNA was adjusted to 18 μl with RNase-free water. The amount of 2 μl of 10X RNA Fragmentation Buffer (100 mM Tris-HCl, 100 mM ZnCl2 in nuclease-free H_2_O) was added and incubated in a preheated thermal cycler for approximately 5 to 6 minutes at 70 °C. The reaction was stopped by adding 2 μl of 0.5 M EDTA. Then added to the mixture was 178 μl of H_2_O, 20 μl of sodium acetate (3 M [pH 5.2]; S7899; Sigma-Aldrich, St. Louis, MO), 14.4 μl of glycogen (5 mg/mL; AM9510; Thermo Fisher Scientific), and 500 μl of 100% ethanol, and the mixture was incubated at −80 °C overnight. Fragmented RNA was pelleted by centrifuge, washed once with 75% ethanol, and resuspended in ultrapure H_2_O (10 μl H_2_O per 1 μg human total RNA). The size distribution of fragmented RNA was assessed using High Sensitivity RNA Screentape on TapeStation (5067–5576; Agilent Technologies; Santa Clara, CA). The total RNA was chemically fragmented into approximately 200-nt-long fragments.

### Spike-in controls for m^6^A MeRIP—*Escherichia coli* K-12

Lyophilized *E*. *coli* K-12 cells were purchased from Sigma-Aldrich (EC1). *E*. *coli* K-12 cells were cultured at 37 °C in LB media with shaking at 280 rpm overnight. Total RNA was extracted using PureLink RNA Mini Kit (12183018A; Thermo Fisher Scientific) according to the manufacturer's protocol. Briefly, the bacterial *E*. *coli* K-12 population was pelleted by centrifuge at 500 × g at 4 °C for 10 minutes and resuspended in lysis buffer prepared with 2-mercaptoethanol. The cell lysate was homogenized by passing approximately 5 to 10 times through an 18- to 21-gauge needle. After centrifugation, the supernatant was collected, after which 250 μl of 100% ethanol was added to the bacterial cell homogenate and mixed together. The mixture was transferred to a Spin Cartridge and centrifuged, washed, and eluted with ultrapure H_2_O. Total RNA extracted from *E*. *coli* K-12 was then DNase treated and further purified using Trizol reagent (Thermo Fisher Scientific) (S5 Fig). For each m^6^A MeRIP experiment, 9 ng of *E*. *coli* K-12 total RNA was added to 2 μg of human total RNA sample to get approximately 1.5% mapping alignment ratio of K-12/human RNA. K-12 total RNA was added to the human total RNA sample before RNA fragmentation. Once K-12 and human RNAs were combined, the sample was treated as a single m^6^A MeRIP throughout the experiment until completion of RNA-seq.

### m^6^A MeRIP

m^6^A MeRIP is based on the previously described m^6^A-seq protocol [27] with several modifications: 30 μl of protein A magnetic beads (10002D; Thermo Fisher Scientific) and 30 μl of protein G magnetic beads (10004D; Thermo Fisher Scientific) were washed twice by IP buffer (150 mM NaCl, 10 mM Tris-HCl [pH 7.5], 0.1% IGEPAL CA-630 in nuclease-free H_2_O), resuspended in 500 μl of IP buffer, and tumbled with 5 μg anti-m^6^A antibody at 4 °C for at least 6 hours. Following 2 washes in IP buffer, the antibody–bead mixture was resuspended in 500 μl of the IP reaction mixture containing fragmented total RNA, 100 μl of 5× IP buffer, and 5 μl of RNasin Plus RNase Inhibitor (N2611; Promega, Madison, WI) and incubated for 2 hours at 4 °C.

In the low/high salt-washing method, the RNA reaction mixture was washed twice in 1,000 μl of IP buffer, twice in 1,000 μl of low-salt IP buffer (50 mM NaCl, 10 mM Tris-HCl [pH 7.5], 0.1% IGEPAL CA-630 in nuclease-free H_2_O), and twice in 1,000 μl of high-salt IP buffer (500 mM NaCl, 10 mM Tris-HCl [pH 7.5], 0.1% IGEPAL CA-630 in nuclease-free H_2_O) for 10 minutes each at 4 °C. After extensive washing, the m^6^A-enriched fragmented RNA was eluted from the beads in 200 μl of RLT buffer supplied in RNeasy Mini Kit (74106; QIAGEN; Germany) for 2 minutes at room temperature. A magnetic separation rack was used to pull beads to the side of the tube. Supernatant was collected to a new tube, and 400 μl of 100% ethanol was added to it. The mixture was transferred to an RNeasy MiniElute spin column and centrifuged at >12,000 rpm at 4 °C for 1 minute. The spin column membrane was washed with 500 μl of RPE buffer once, then 500 μl of 80% ethanol once, and centrifuged at full speed for 5 minutes at 4 °C to remove the residual ethanol. The m^6^A-enriched RNA was eluted with 14 μl ultrapure H_2_O. For a second round of IP, eluted RNA was re-incubated with protein A/G magnetic beads coupled to anti-m^6^A antibody, followed by washes, elution from the protein A/G beads, and purification as above. In addition, it is the high salt that contributes to better S/N ratio (S7A Fig).

In the m^6^A competitive elution method, the m^6^A competitive elution buffer for each pulldown was prepared by mixing 45 μl of 5× IP buffer, 75 μl of 20 mM m^6^A (M2780; Sigma-Aldrich), 3.5 μl of RNasin Plus RNase Inhibitor, and 101.5 μl of ultrapure H_2_O. The immunoprecipitated m^6^A RNA with protein A/G magnetic beads was then washed 3 times in 1,000 μl of IP buffer for 10 minutes each at 4 °C and was resuspended in 100 μl of m^6^A competitive elution buffer with continuous shaking for 1 hour at 4 °C. The mixture was placed on a magnetic separation rack, and supernatant containing the eluted m^6^A RNA was collected to a new tube. Then, another 100 μl of m^6^A competitive elution buffer was added for one more elution. To purify the eluted RNA, 700 μl of RLT buffer and 1,400 μl of 100% ethanol were added to 200 μl of eluted supernatant collected and mixed thoroughly. The mixture was transferred to an RNeasy MiniElute spin column (QIAGEN) and centrifuged at >12,000 rpm at 4 °C for 1 minute. This step was repeated until all of the sample was loaded to the column. The spin column membrane was washed with 500 μl of RPE buffer once, then 500 μl of 80% ethanol once, and centrifuged at full speed for 5 minutes at 4 °C to remove the residual ethanol. The m^6^A-enriched RNA was eluted with 14 μl ultrapure H_2_O.

The MeRIP-seq data has been deposited in NCBI GEO database (GSE116002).

### m^6^A real-time qPCR

cDNA was synthesized from total RNA using the High-Capacity cDNA Reverse Transcription Kit (4368814; Thermo Fisher Scientific). Gluc, Cluc, *SETD7*, and *GAPDH* genes were amplified using the primers listed below: Gluc forward primer: 5´- CGACATTCCTGAGATTCCTGG—3´; GLuc reverse primer: 5´- TTGAGCAGGTCAGAACACTG—3´; CLuc forward primer: 5´- GCTTCAACATCACCGTCATTG—3´; CLuc reverse primer: 5´- CACAGAGGCCAGAGATCATTC—3´; *SETD7* forward primer: 5´- GGGGTTCAGAGACCTGGAAT—3´; *SETD7* reverse primer: 5´- GCATGGTGAGAGGATGTGAC—3´; *GAPDH* forward primer: 5´- TCAAGGCTGAGAACGGGAAG—3´; *GAPDH* reverse primer: 5´- GGACTCCACGACGTACTCAG—3´. The following was calculated to determine the expression percentage of a target gene in IP sample relative to that in input sample: %Input = 2^(Ct of target gene in IP sample − Ct of target gene in input sample). The S/N ratio was calculated relative to the negative region detected with CLuc or *GAPDH* using the following formula: S/N ratio = %Input of positive region (GLuc or *SETD7*) ÷ %Input of negative region (CLuc or *GAPDH*). The experiment was repeated 3 times independently.

### Library preparation

The amount of 2 μl of 14 μl eluted RNA was reverse transcribed with High-Capacity cDNA Reverse Transcription Kit (Thermo Fisher Scientific). IP efficiency was assessed by GLuc/CLuc or *SETD7*/*GAPDH* real-time PCR. Once successfully immunoprecipitated methylated RNA was confirmed, further transcriptome-wide interrogation was pursued by deep sequencing using SMARTer Stranded Total RNA-Seq Kit version 2 (Pico Input Mammalian; 634413; Takara/Clontech, Japan) according to the manufacturer's protocol. Briefly, 3.5 μl of 14 μl eluted RNA and 50 ng input RNA were used for library construction, entering the protocol without fragmentation by adding first-strand cDNA synthesis mix. From that point on, the exact steps of the SMARTer Stranded Total RNA-Seq Kit version 2 (Pico Input Mammalian) user manual were followed to the end. Libraries for IP RNA were PCR amplified for 16 cycles, whereas 12 cycles were used for input RNA. Optimal for an m^6^A IP sample derived from 2 μg total RNA is 16 amplification cycles (S7B Fig). The optimal cycle number needs to be determined for samples with different starting RNA amounts. A purified library was quantified using a Qubit Fluorometer (Thermo Fisher Scientific), and the size distribution was checked using TapeStation D1000 ScreenTape (Agilent Technologies). The samples were then sequenced using a NextSeq 500 High Output Mode 75-cycle kit (Illumina, San Diego, CA) as single ends. Adapter sequences were removed, and sequences were demultiplexed using the bcl2fastq version 2 software (Illumina).

### METTL3 knockdown by siRNA

A549 cells were transfected with 50 nM siMETTL3_1 (sense: 5’-CCUGCAAGUAUGUUCACUATT-3’), siMETTL3_2 (sense: 5’-GCUCAACAUACCCGUACUATT-3’) (RiboBio, Guangzhou, China), or control siRNA (siControl) (sense: 5’- UUCUCCGAACGUGUCACGUTT-3’) (RiboBio) using lipofectamine 2000 (Invitrogen) according to the manufacturer’s protocols.

### RNA extraction and real-time PCR

Total RNA was extracted using Trizol reagent (Invitrogen, Carlsbad, CA). cDNA was synthesized from total RNA using Transcriptor Reverse Transcriptase (Roche Applied Sciences, Indianapolis, IN). *METTL3* (forward primer: 5’-CCACTGATGCTGTGTCCATC-3’; reverser primer: 5’-GGAGACCTCGCTTTACCTCA-3’) and *SLC2A1* (forward primer: 5’-CCAGCAGCAAGAAGCTGAC-3’; reverser primer: 5’-TGGACCCATGTCTGGTTGTA-3’) genes were amplified with β-actin (forward primer: 5’-GTCTTCCCCTCCATCGTG-3’; reverser primer: 5’- AGGGTGAGGATGCCTCTCTT-3’) as an internal control.

### Western blot

Total protein was extracted from cell pellets. Thirty micrograms of protein from each sample were separated on 12% SDS-PAGE and transferred onto nitrocellulose membranes (GE Healthcare, Piscataway, NJ). Blots were immunostained with primary antibody (*METTL3*: number 96391, Cell Signaling Technology, Danvers, MA; *SLC2A1*: ab15309, Abcam, UK; Flag: F1804, Sigma-Aldrich) and secondary antibody, respectively. *GAPDH* or β-ACTIN was used as a loading control.

### Transcriptomic and proteomic profile of human lung ADC patients

RNA-seq of ADC patients was performed using the Illumina TruSeq RNA Sample Prep Kit version 2. Proteomic profiles of human ADC tumors were generated using super-SILAC and label-free quantification (LFQ) approaches as described previously [46]. Super-SILAC standard was added to 30 μg protein lysate from each tumor sample. Protein lysates were processed using the filter-aided sample preparation method. The eluted peptides were dissolved in 0.1% formic acid for and Liquid chromatography-tandem mass spectrometry (LC-MS/MS) analysis. The raw MS data were analyzed by MaxQuant [47], and MS/MS spectra were searched against the UniProt human database (http://www.uniprot.org) using the Andromeda search engine. Finally, the LFQ strategies were used to determine the protein relative abundance [46].

### Published m^6^A datasets

For comparing the performance of MACS and MeTPeak, 2 publicly available m^6^A-seq datasets were used [22,27].

### Alignment and reads coverage

MeRIP-seq reads were aligned to human genome hg38 by using the STAR (version 2.4.2a) [48] with reference annotation GENCODE [49] version 25. For samples with *E*. *coli* K-12 spike-in, all spike-in sequences were incorporated with the human genome. To minimize the rate of false positives, only uniquely mapped and without-duplication reads were selected by samtools (version 1.3.1) [50] and were separated by strand for those strand-specific samples with RSeQC (version 2.6.1) [51] (alignment summary: S1, S2 and S5 Tables). Then, the reads were extended to a length of 200 nt in the 5’-to-3’ direction, accounting for the length of RNA fragments, to calculate the reads coverage along the genome by MACS [32] subcommand pileup. To facilitate the reads coverage visualization and comparison among different samples, UCSC tools [52] and RSeQC [51] were employed for bigwig format transforming and normalization separately. RNA-seq reads were aligned to hg38 with reference annotation GENCODE version 25 by STAR [48]. HTSeq [53] plus DESeq2 [54] pipeline was used to quantify and normalize the mRNA expression level.

### m^6^A site detection and characterization

For genome-based peak caller MACS, the effective genome size was set to be 3.0 × 10^8^, and—*shiftsize* was set based on the length of RNA fragments under the option of—*nomodel*; the summit for m^6^A peaks is the location with the highest IP fragment pileup [32]. For transcriptome-based peak caller MeTPeak [35], the m^6^A peak region summit was defined as the site with the highest fragment pileup ratio between the IP and Input. The top 5,000 peaks were chosen for de novo motif analysis with DREME [55], which takes 200-nt-long peak summit-centered sense sequences as input. Peaks falling in mRNA were assigned to 5 nonoverlapped regions, which are TSS downstream 200 nt, 5’UTR, CDS, stop-codon–centered 400 nt, and 3’UTR.

### Differential m^6^A level analysis

First of all, the m^6^A peaks from any 2 samples were classified as unique and common based on whether they overlapped or not. Then, the common peaks between samples were merged and combined with unique ones to be the reference m^6^A peaks list. After, the reads for IP and Input after removal of duplication were subsampled to the same read depth, and the extent reads were counted for each peak under IP and Input separately. In addition, the read counts were further corrected based on the total number of ERCC spike-in reads (S5 Table) to remove other latent technical biases (S6B and S6C Fig). Then, based on the contingency table of 2 samples by IP/Input read counts for each peak, a Fisher exact test was employed to test whether the m^6^A levels were significantly different or not (*p* < 0.05) (S6B-D). Furthermore, highly differential m^6^A peaks were selected with additional constraint abs(log2[R_Tumor1_/R_Tumor2_]) > 1 (Fig 5C), and those genes with only 1 highly differential m^6^A peak were collected to check the correlation between the mRNA and protein based on RNA-seq data and MS data.

## Supporting information

S1 Fig Calibration of RNA fragmentation and validation of size distribution by Agilent TapeStation System. (A) Different RNA samples were chemically fragmented for the specified time points, ethanol-precipitated, and separated on the High Sensitivity RNA ScreenTape. After 6 minutes, RNA fragments centered around approximately 200 nt. (B, C) Representative electropherogram of fragmented RNA using 5 μg total RNA from A549 cells after 6- and 7-minute incubation.
(TIFF)

S2 Fig Comparison between MACS and MeTPeak using the HepG2 MeRIP-seq dataset. (A) Peaks detected by MACS and MeTPeak with and without the duplication reads. (B) Changes in peak number with increasing depth of uniquely mapped reads for IP/Input. The dashed line means the overlapped peaks called by both MeTpeak and MACS. (C) Consistent m^6^A motifs detected from top 5,000 m^6^A summit centered around 200 nt regions. (D) The location and frequency of the top motif to the summit; ***p < 1 × 10^−4^. (E) The distribution characteristic of the peaks detected by MACS based on 2 published m^6^A datasets. (F) Motif discovered by MACS based on 2 published m^6^A datasets. Data related to this figure can be found in S1 Data. IP, immunoprecipitation; m^6^A, N6-methyladenosine; MeRIP-seq, m^6^A RNA immunoprecipitation followed by high-throughput sequencing.
(TIFF)

S3 Fig Peak distribution and motif analyses of m^6^A MeRIP-seq. (A) Comparison between the control dataset (300 μg) and the Millipore or Synaptic 32 μg library data. Left: Venn diagrams show the overlap between m^6^A peaks from different libraries. Right: the motif called based on Millipore or Synaptic 32 μg unique peaks. (B) Analysis of the percentage of m^6^A peaks in each of the 5 nonoverlapping transcript segments. (C) Relative enrichment of m^6^A peaks around the TSS and stop codon region. (D) Sequence logo representing the deduced top motifs for 12 μg and 6 μg libraries. (E) Density curves of the motif flanking the peak summit. (F, G) Comparison between the Millipore 2 μg and the control dataset. Data related to this figure can be found in S1 Data. MeRIP-seq, m^6^A RNA immunoprecipitation followed by high-throughput sequencing; TSS, transcription start site.
(TIFF)

S4 Fig Reproducibility of MeRIP-seq and optimization of antibody/RNA ratio. (A) The S/N ratio of *SETD7*/*GAPDH* with different starting amount of total RNA. (B) Correlation between 2 replicates for both Input (top) and IP (bottom) of the 0.5 μg MeRIP-seq data. (C) The S/N ratio of *SETD7*/*GAPDH* (top) and the *SETD7* IP yield (percentage of the input) (bottom) using different amounts of Millipore antibody (5 μg, 1 μg, and 0.2 μg) with fixed amount of total RNA (2 μg). Data related to this figure can be found in S1 Data. IP, immunoprecipitation; MeRIP-seq, m^6^A RNA immunoprecipitation followed by high-throughput sequencing; S/N, signal-to-noise.
(TIFF)

S5 Fig *E*. *coli* K-12 spiked in for quality control. (A) The gel image separation profile of *E*. *coli* K-12 total RNA before and after DNase treatment on the High Sensitivity RNA ScreenTape. (B) Representative electropherogram of *E*. *coli* K-12 total RNA before and after purification and DNase treatment.
(TIFF)

S6 Fig ERCC spike-in based normalization. (A) Venn diagram of peaks detected in 2 ADC tumors (tumor1 and tumor2). (B) The correlation between IP fold change and Input fold change before the ERCC spike-in normalization (top). MA plots for different m^6^A peaks before normalization (bottom). (C) The correlation between IP fold change and Input fold change (top). MA plots for different m^6^A peaks after normalization were shown in the bottom. (D) Number of differential peaks distributed along the log-transformed fold change. Data related to this figure can be found in S1 Data. ADC, adenocarcinoma; IP, immunoprecipitation; MA, M is the binary logarithm of the intensity ratio and A is the average log intensity.
(TIFF)

S7 Fig Additional refinement of m^6^A MeRIP-seq. (A) Comparison of low-salt, high-salt, and low/high salt combination washing conditions. Top: pulldown efficiency as measured by S/N ratio of *SETD7*/*GAPDH*. Bottom: *SETD7* IP yield (percentage of the input). (B) MeRIP-seq library of different amplification cycles using SMARTer Stranded Total RNA-Seq Kit version 2 (Pico Input Mammalian) kit. One out of 50 μl of PCR product was used for gel electrophoresis by DNA tape station. The smear centered at 300 bp is the library DNA. IP, immunoprecipitation; MeRIP-seq, m^6^A RNA immunoprecipitation followed by high-throughput sequencing; S/N, signal-to-noise.
(TIFF)

S1 Table Summary of m6A MeRIP-seq data in A549 with different starting RNA amounts. MeRIP-seq, m^6^A RNA immunoprecipitation followed by high-throughput sequencing.
(XLSX)

S2 Table Summary of m6A MeRIP-seq data starting with 0.5 μg RNA in A549. MeRIP-seq, m^6^A RNA immunoprecipitation followed by high-throughput sequencing.
(XLSX)

S3 Table The m6A peak distribution in protein coding gene and lincRNA. lincRNA, long intergenic noncoding RNA; m^6^A, N6-Methyladenosine.
(XLSX)

S4 Table Overlap between MeRIP-seq peaks and single nucleotide resolution m6A sites. m^6^A, N6-Methyladenosine; MeRIP-seq, m^6^A RNA immunoprecipitation followed by high-throughput sequencing.
(XLSX)

S5 Table Summary of m6A MeRIP-seq data from tumor1 and tumor2 with ERCC spike-in. MeRIP-seq, m^6^A RNA immunoprecipitation followed by high-throughput sequencing.
(XLSX)

S1 Text The refined MeRIP-seq protocol. MeRIP-seq, m^6^A RNA immunoprecipitation followed by high-throughput sequencing.
(DOCX)

S1 Data All raw data used for quantification in this work. (XLSX)

## Abbreviations

1–200AA: 1–200 amino acids
ADC: adenocarcinoma
ATCC: American Type Culture Collection
CDS: coding sequence
ChIP-seq: chromatin immunoprecipitation followed by DNA sequencing
EGFR: Epidermal Growth Factor Receptor
IP: immunoprecipitation
LC-MS/MS: Liquid chromatography-tandem mass spectrometry
LFQ: label-free quantification
lincRNA: long intergenic noncoding RNA
lncRNA: long noncoding RNA
m^6^A: N6-Methyladenosine
m^6^A MeRIP-seq: m^6^A RNA immunoprecipitation followed by high-throughput sequencing
METTL3: Methyltransferase Like 3
miRNA: microRNA
MS: mass spectrometry
NEB: New England Biolabs
PDX: patient-derived xenograft
RNA-seq: RNA sequencing
RPKM: Reads Per Kilobase of transcript per Million mapped reads
RT-qPCR: quantitative reverse transcription PCR
SILAC: Stable Isotope Labeling by Amino acids in Cell culture
siRNA: small interfering RNA
SCARLET: Site-specific cleavage and radioactive labeling followed by ligation-assisted extraction and thin-layer chromatography
SLC2A1: Solute Carrier family 2, Facilitated Glucose Transporter member 1
S/N: signal-to-noise
SySy: Synaptic Systems
TSS: transcription start site
UTR: untranslated region
