# Supplementary figures and images for: Refined RIP-seq protocol for epitranscriptome analysis with low input materials
Source: PLoS Biol. 2018 Sep 13;16(9):e2006092. doi: 10.1371/journal.pbio.2006092 (PMC6136692; doi:10.1371/journal.pbio.2006092)

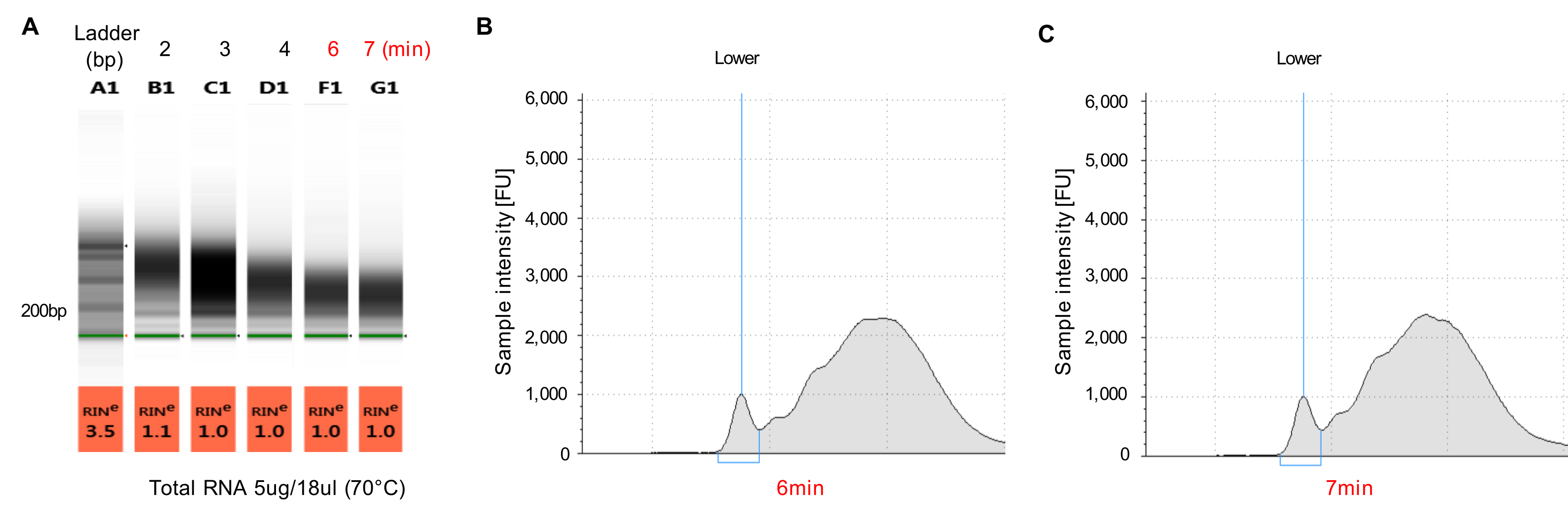

Supplement: S1 Fig — (A) Different RNA samples were chemically fragmented for the specified time points, ethanol-precipitated, and separated on the High Sensitivity RNA ScreenTape. After 6 minutes, RNA fragments centered around approximately 200 nt. (B, C) Representative electropherogram of fragmented RNA using 5 μg total RNA from A549 cells after 6- and 7-minute incubation. (TIFF) [file pbio.2006092.s001.tiff]

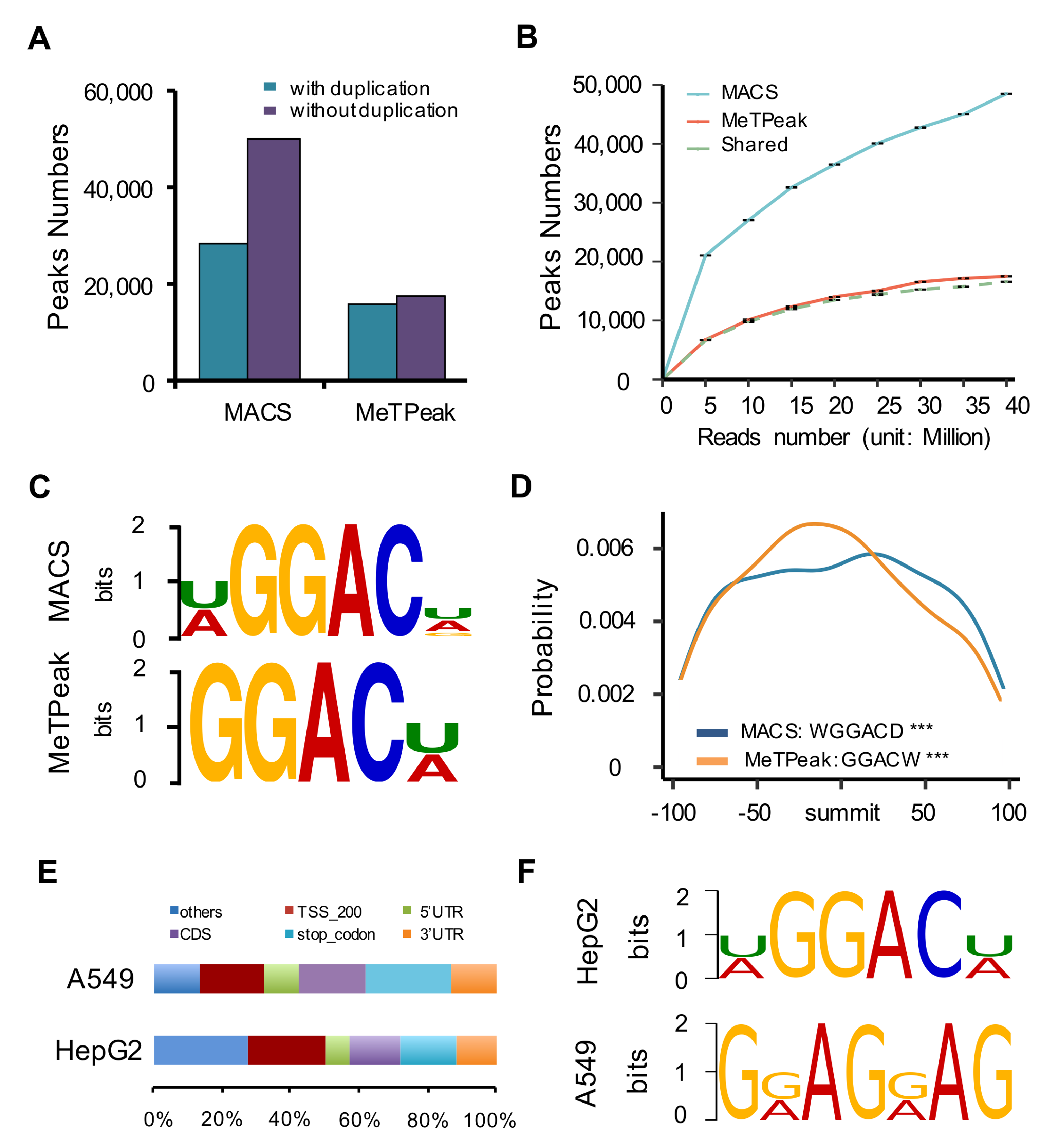

Supplement: S2 Fig — (A) Peaks detected by MACS and MeTPeak with and without the duplication reads. (B) Changes in peak number with increasing depth of uniquely mapped reads for IP/Input. The dashed line means the overlapped peaks called by both MeTpeak and MACS. (C) Consistent m6A motifs detected from top 5,000 m6A summit centered around 200 nt regions. (D) The location and frequency of the top motif to the summit; ***p < 1 × 10^−4^. (E) The distribution characteristic of the peaks detected by MACS based on 2 published m6A datasets. (F) Motif discovered by MACS based on 2 published m6A datasets. Data related to this figure can be found in S1 Data. IP, immunoprecipitation; m6A, N6-methyladenosine; MeRIP-seq, m6A RNA immunoprecipitation followed by high-throughput sequencing. (TIFF) [file pbio.2006092.s002.tiff]

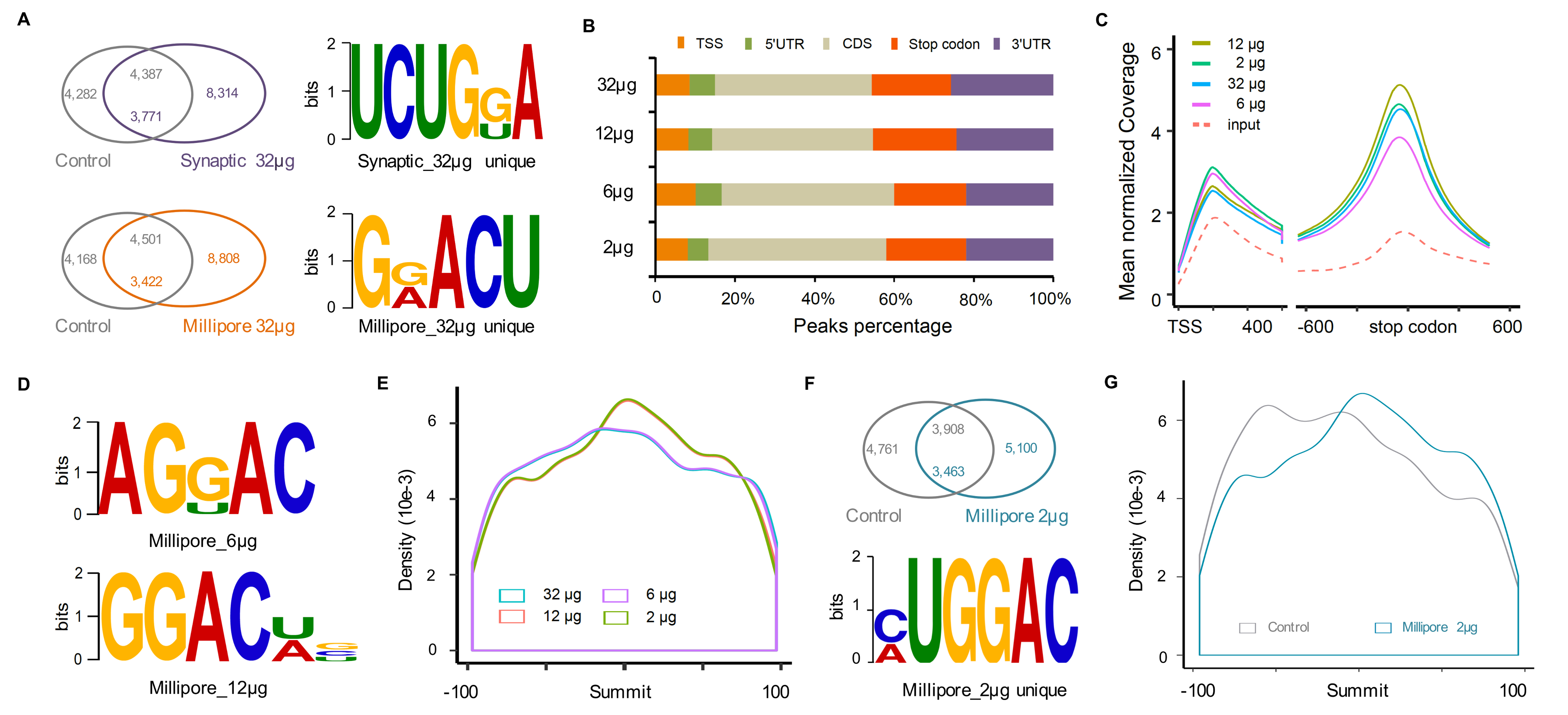

Supplement: S3 Fig — (A) Comparison between the control dataset (300 μg) and the Millipore or Synaptic 32 μg library data. Left: Venn diagrams show the overlap between m6A peaks from different libraries. Right: the motif called based on Millipore or Synaptic 32 μg unique peaks. (B) Analysis of the percentage of m6A peaks in each of the 5 nonoverlapping transcript segments. (C) Relative enrichment of m6A peaks around the TSS and stop codon region. (D) Sequence logo representing the deduced top motifs for 12 μg and 6 μg libraries. (E) Density curves of the motif flanking the peak summit. (F, G) Comparison between the Millipore 2 μg and the control dataset. Data related to this figure can be found in S1 Data. MeRIP-seq, m6A RNA immunoprecipitation followed by high-throughput sequencing; TSS, transcription start site. (TIFF) [file pbio.2006092.s003.tiff]

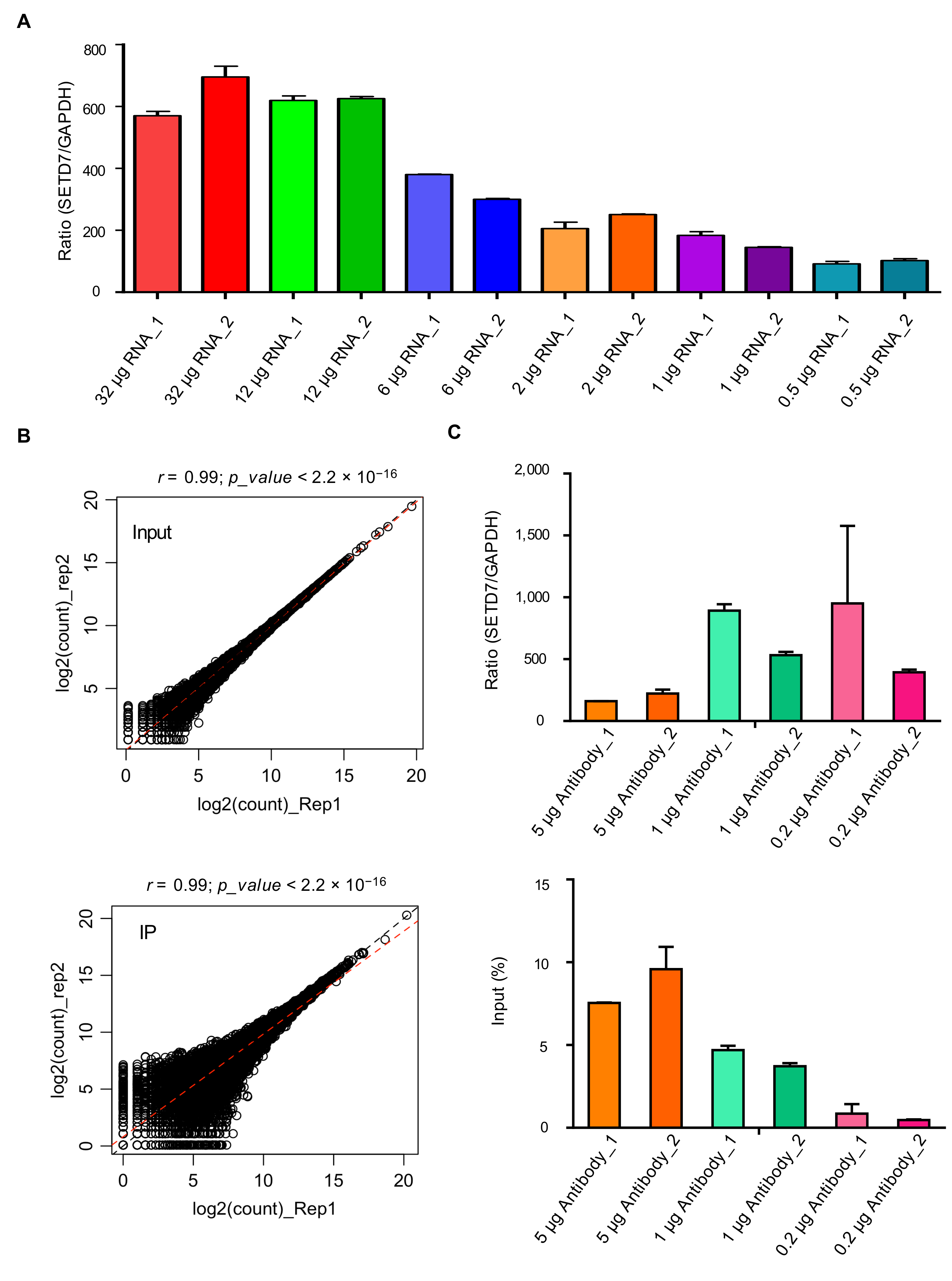

Supplement: S4 Fig — (A) The S/N ratio of SETD7/GAPDH with different starting amount of total RNA. (B) Correlation between 2 replicates for both Input (top) and IP (bottom) of the 0.5 μg MeRIP-seq data. (C) The S/N ratio of SETD7/GAPDH (top) and the SETD7 IP yield (percentage of the input) (bottom) using different amounts of Millipore antibody (5 μg, 1 μg, and 0.2 μg) with fixed amount of total RNA (2 μg). Data related to this figure can be found in S1 Data. IP, immunoprecipitation; MeRIP-seq, m6A RNA immunoprecipitation followed by high-throughput sequencing; S/N, signal-to-noise. (TIFF) [file pbio.2006092.s004.tiff]

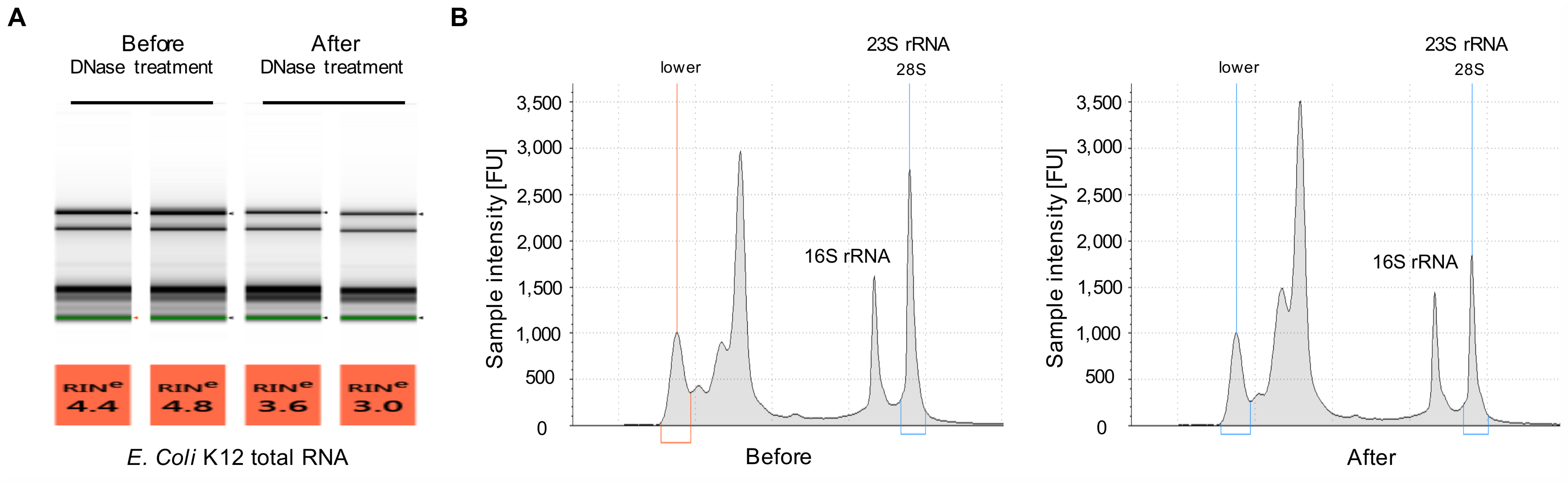

Supplement: S5 Fig — (A) The gel image separation profile of E. coli K-12 total RNA before and after DNase treatment on the High Sensitivity RNA ScreenTape. (B) Representative electropherogram of E. coli K-12 total RNA before and after purification and DNase treatment. (TIFF) [file pbio.2006092.s005.tiff]

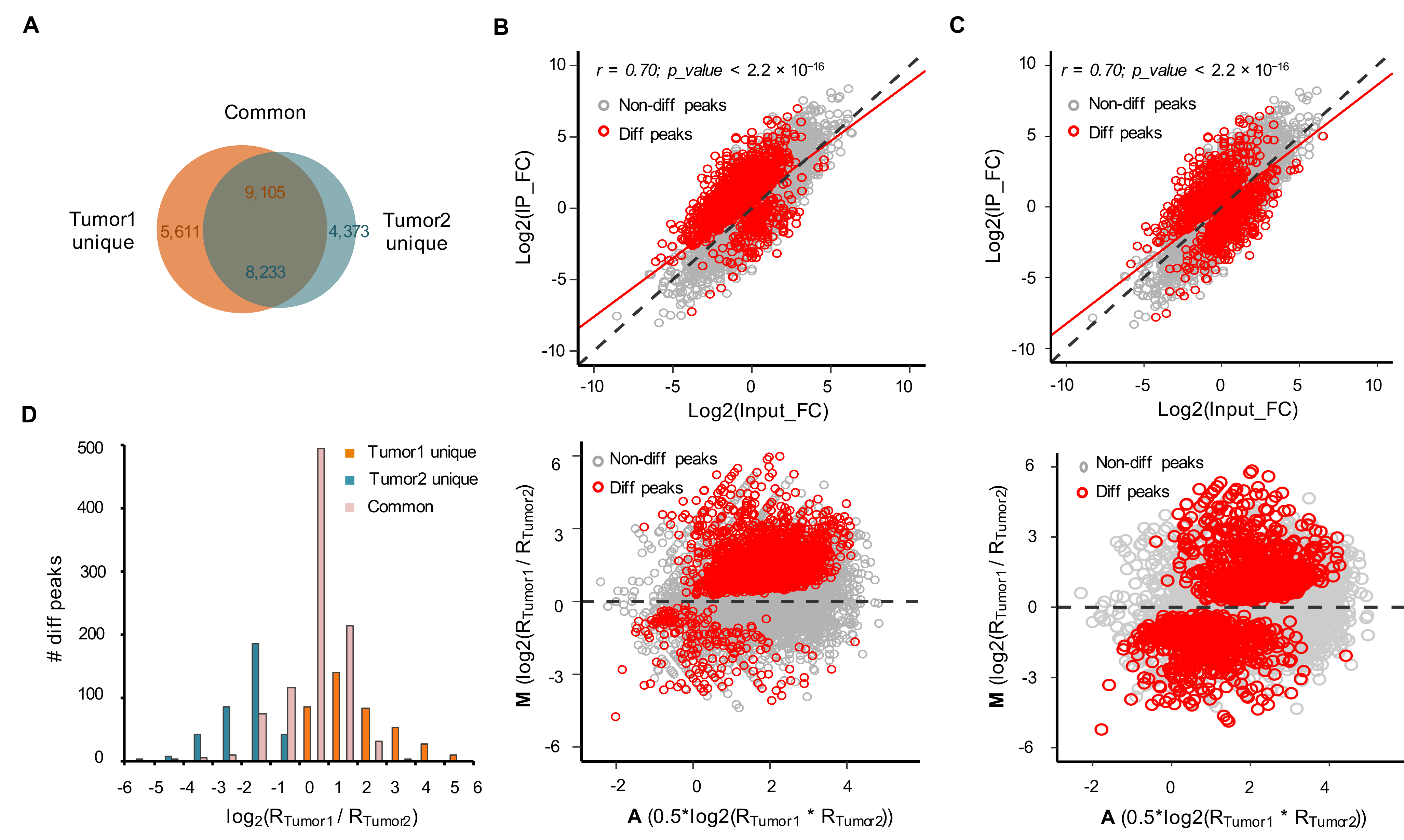

Supplement: S6 Fig — (A) Venn diagram of peaks detected in 2 ADC tumors (tumor1 and tumor2). (B) The correlation between IP fold change and Input fold change before the ERCC spike-in normalization (top). MA plots for different m6A peaks before normalization (bottom). (C) The correlation between IP fold change and Input fold change (top). MA plots for different m6A peaks after normalization were shown in the bottom. (D) Number of differential peaks distributed along the log-transformed fold change. Data related to this figure can be found in S1 Data. ADC, adenocarcinoma; IP, immunoprecipitation; MA, M is the binary logarithm of the intensity ratio and A is the average log intensity. (TIFF) [file pbio.2006092.s006.tiff]

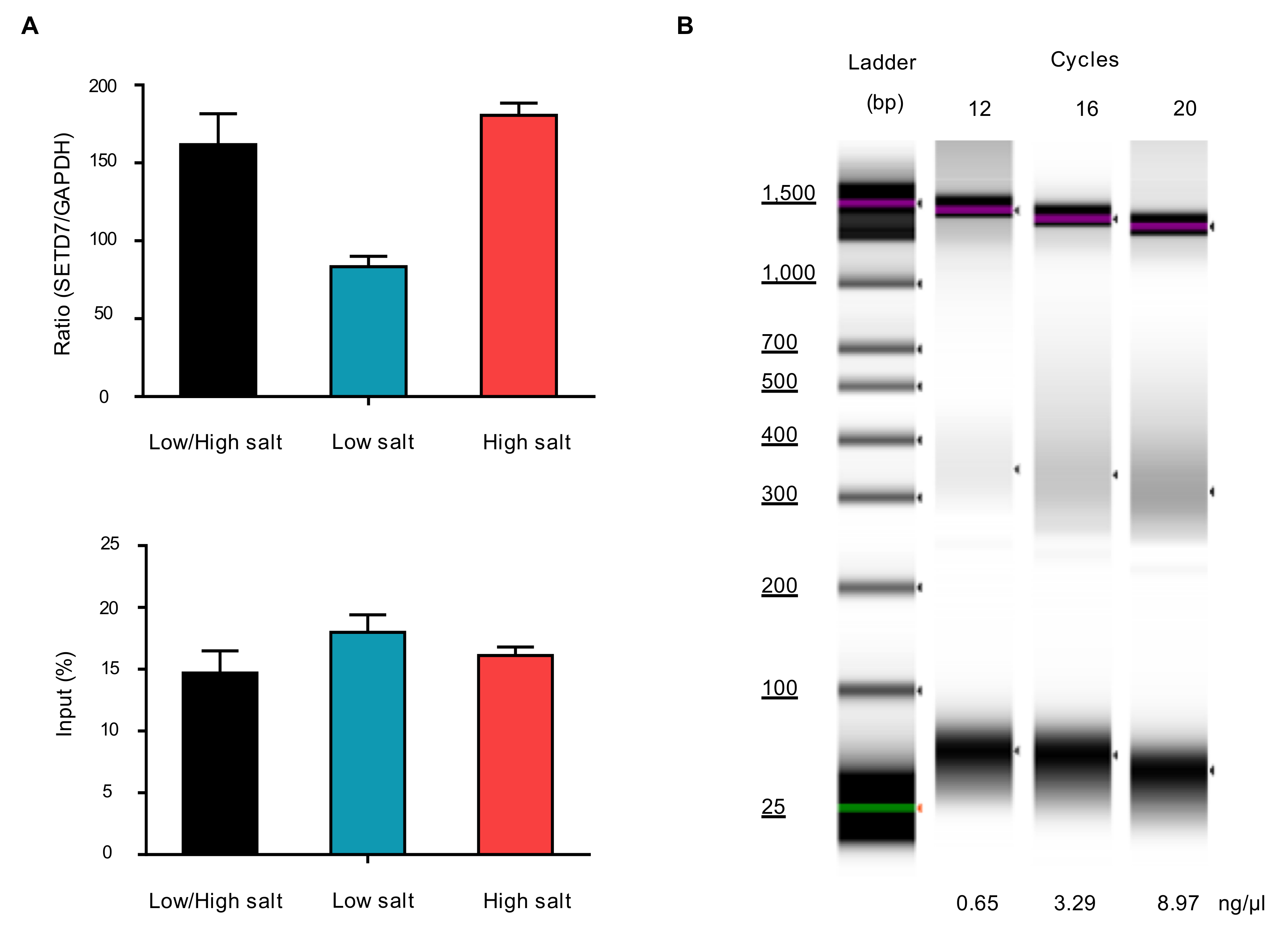

Supplement: S7 Fig — (A) Comparison of low-salt, high-salt, and low/high salt combination washing conditions. Top: pulldown efficiency as measured by S/N ratio of SETD7/GAPDH. Bottom: SETD7 IP yield (percentage of the input). (B) MeRIP-seq library of different amplification cycles using SMARTer Stranded Total RNA-Seq Kit version 2 (Pico Input Mammalian) kit. One out of 50 μl of PCR product was used for gel electrophoresis by DNA tape station. The smear centered at 300 bp is the library DNA. IP, immunoprecipitation; MeRIP-seq, m6A RNA immunoprecipitation followed by high-throughput sequencing; S/N, signal-to-noise. (TIFF) [file pbio.2006092.s007.tiff]
