# Supplementary material for: Refined RIP-seq protocol for epitranscriptome analysis with low input materials
Source: PLoS Biol. 2018 Sep 13;16(9):e2006092. doi: 10.1371/journal.pbio.2006092 (PMC6136692; doi:10.1371/journal.pbio.2006092)
Supplement: S1 Text — MeRIP-seq, m6A RNA immunoprecipitation followed by high-throughput sequencing. (DOCX) [file pbio.2006092.s013.docx]

**Procedure**

**RNA isolation**

**Step 1:** Total RNA from cells in culture was extracted using Trizol reagent (15596018, Thermo Fisher Scientific, Waltham, MA).

**Step 2:** Total RNA was treated with DNase I (04 716 728 001, Roche Diagnostics, Indianapolis, IN) for 20 min at 37°C to remove DNA contamination.

**Step 3:** The RNA was precipitated using glycogen (25 µg/mL final) (5 mg/ml, AM9510, Thermo Fisher Scientific) and isopropanol at −30°C for 2 hrs. The precipitated RNA was then washed with 70% ethanol. The final pellet was resuspended in ultrapure H_2_O.

**Step 4:** The concentration of total RNA was measured by Qubit RNA HS Assay Kit (Q32855, Thermo Fisher Scientific).

**Preparation of spike-in controls - *E. coli* K-12**

**Step 1:** Lyophilized *E. coli* K-12 cells were purchased from Sigma-Aldrich (EC1). *E. coli* K-12 cells were cultured at 37°C in LB media with shaking at 280 rpm overnight.

**Step 2:** Total RNA was extracted using PureLink RNA Mini Kit (12183018A, Thermo Fisher Scientific). *E. coli* K-12 population was pelleted by centrifuge at 500 × g at 4°C for 10 min and resuspended in Lysis Buffer prepared with 2-mercaptoethanol. The cell lysate was homogenized by passing 5 ~ 10 times through an 18-21-gauge needle. After centrifuge, the supernatant was collected.

**Step 3:** Then 250 µl of 100% ethanol was added to the bacterial cell homogenate and mixed. The mixture was transferred to a Spin Cartridge and centrifuged, washed and eluted with ultrapure H_2_O.

**Step 4:** Total RNA extracted from *E. coli* K-12 was then DNase-treated and further purified using Trizol reagent (Thermo Fisher Scientific) (S5 Fig).

**Step 5:** For each m6A MeRIP experiment, 9 ng *E. coli* K-12 total RNA was added to 4 µg human total RNA sample to get ~ 1.5% mapping alignment ratio of K12/Human RNA. K-12 total RNA was added to human total RNA sample before RNA fragmentation. Once K-12 and human RNAs were combined, the sample was treated as a single m^6^A MeRIP throughout the experiment until completion of RNA sequencing.

**RNA fragmentation**

**Step 1:** The total volume of 3 ~ 5 µg total RNA was adjusted to 18 µl with RNase-free water.

**Step 2:** 2 µl of 10X RNA Fragmentation Buffer (100 mM Tris-HCl, 100 mM ZnCl2 in nuclease free H_2_O) was added and incubated in a preheated thermal cycler for 5 ~ 6 min at 70°C.

**Step 3:** The reaction was stopped by adding 2 µl of 0.5 M EDTA. The mixture was then added with 178 µl of H_2_O, 20 µl of sodium acetate (3 M, pH 5.2, S7899, Sigma-Aldrich, St. Louis, MO), 14.4 µl of glycogen (5 mg/ml, AM9510, Thermo Fisher Scientific) and 500 µl of 100% ethanol and incubated at −80°C overnight.

**Step 4:** Fragmented RNA was pelleted by centrifuge, washed once with 75% ethanol and resuspended in ultrapure H_2_O (10 µl H_2_O per 1 µg human total RNA).

**Step 5:** The size distribution of fragmented RNA was assessed using High Sensitivity RNA Screentape on TapeStation (5067-5576, Agilent Technologies, Santa Clara, CA). The total RNA was chemically fragmented into ∼200-nt-long fragments.

**m^6^A MeRIP**

**Step 1:** 30 µl of protein-A magnetic beads (10002D, Thermo Fisher Scientific) and 30 µl of protein-G magnetic beads (10004D, Thermo Fisher Scientific) were washed twice by IP buffer (150 mM NaCl, 10 mM Tris-HCl, pH 7.5, 0.1% IGEPAL CA-630 in nuclease free H_2_O) and resuspended in 500 μl of IP buffer, and tumbled with 5 μg anti-m^6^A antibody at 4°C for at least 6 hrs. We used the following antibodies against m^6^A: rabbit polyclonal anti-m^6^A (202 003, Synaptic Systems, Germany; ABE572, Millipore, Germany) and rabbit monoclonal anti-m^6^A supplied in EpiMark N6-Methyladenosine Enrichment Kit (E1610S, NEB, Ipswich, MA). Among these antibodies, antibody from Millipore (ABE572) gave the best efficiency and specificity.

**Step 2:** Following 2 washes in IP buffer, the antibody-bead mixture was resuspended in 500 μl of the IP reaction mixture containing fragmented total RNA, 100 µl of 5X IP buffer and 5 µl of RNasin Plus RNase Inhibitor (N2611, Promega, Madison, WI), and incubated for 2 hrs at 4°C.

**m^6^A MeRIP-In low/high salt washing method:**

**Step 3:** The RNA reaction mixture was then washed twice in 1000 μl of IP buffer, twice in 1000 μl of low-salt IP buffer (50 mM NaCl, 10 mM Tris-HCl, pH 7.5, 0.1% IGEPAL CA-630 in nuclease free H_2_O), and twice in 1000 μl of high-salt IP buffer (500 mM NaCl, 10 mM Tris-HCl, pH 7.5, 0.1% IGEPAL CA-630 in nuclease free H_2_O) for 10 min each at 4°C.

**Step 4:** After extensive washing, the m^6^A enriched fragmented RNA was eluted from the beads in 200 μl of RLT buffer supplied in RNeasy Mini Kit (74106, QIAGEN, Germany) for 2 min at room temperature. Magnetic separation rack was applied to pull beads to the side of the tube. Supernatant was collected to a new tube and added with 400 μl of 100% ethanol. The mixture was transferred to an RNeasy MiniElute spin column and centrifuged at >12000 rpm at 4°C for 1 min. The spin column membrane was washed with 500 μl of RPE buffer once, then 500 μl of 80% ethanol once and centrifuged at full speed for 5 min at 4°C remove the residual ethanol. The m^6^A enriched RNA was eluted with 14 μl ultrapure H_2_O.

**Optional:** For a second round of IP, eluted RNA was re-incubated with protein-A/G magnetic beads coupled to anti-m^6^A antibody, followed by washes, elution from the protein-A/G beads and purification as above.

**m^6^A MeRIP-In m^6^A competitive elution method:**

**Step 3:** The immunoprecipitated m^6^A RNA with protein-A/G-magnetic beads was then washed three times in 1000 μl of IP buffer for 10 min each at 4°C and was resuspended in 100 μl of m^6^A competitive elution buffer with continuous shaking for 1 h at 4°C. The m^6^A competitive elution buffer for each pulldown was prepared by mixing 45 μl of 5X IP buffer, 75 μl of 20 mM m^6^A (M2780, Sigma-Aldrich), 3.5 μl of RNasin Plus RNase Inhibitor and 101.5 μl of ultrapure H_2_O.

**Step 4:** The mixture was placed on magnetic separation rack and supernatant containing the eluted m^6^A RNA was collected to a new tube.

**Step 5:** Then another 100 μl of m^6^A competitive elution buffer was added for one more elution.

**Step 6:** To purify the eluted RNA, 700 μl of RLT buffer and 1400 μl of 100% ethanol were added to 200 μl of eluted supernatant collected and mixed thoroughly. The mixture was transferred to an RNeasy MiniElute spin column (QIAGEN) and centrifuged at >12000 rpm at 4°C for 1 min. This step was repeated until all sample was loaded to the column. The spin column membrane was washed with 500 μl of RPE buffer once, then 500 μl of 80% ethanol once and centrifuged at full speed for 5 min at 4°C remove the residual ethanol. The m^6^A enriched RNA was eluted with 14 μl ultrapure H_2_O.

**m^6^A MeRIP validation using m^6^A quantitative real time PCR**

**Step 1:** cDNA was synthesized from total RNA using High-Capacity cDNA Reverse Transcription Kit (4368814, Thermo Fisher Scientific).

**Step 2:** *Gluc*, *Cluc*, *SETD7* and *GAPDH* gene was ampliﬁed using primers listed below: *Gluc* forward primer: 5´- CGACATTCCTGAGATTCCTGG - 3´; *GLuc* reverse primer: 5´- TTGAGCAGGTCAGAACACTG - 3´; *CLuc* forward primer: 5´- GCTTCAACATCACCGTCATTG - 3´; *CLuc* reverse primer: 5´- CACAGAGGCCAGAGATCATTC - 3´; *SETD7* forward primer: 5´- GGGGTTCAGAGACCTGGAAT - 3´; *SETD7* reverse primer: 5´- GCATGGTGAGAGGATGTGAC - 3´; *GAPDH* forward primer: 5´- TCAAGGCTGAGAACGGGAAG - 3´; *GAPDH* reverse primer: 5´- GGACTCCACGACGTACTCAG - 3´. The following was calculated to determine the expression percentage of a target gene in IP sample relative to that in input sample: %Input = 2^(Ct of target gene in IP sample − Ct of target gene in input sample). The S/N ratio was calculated relative to the negative region detected with *CLuc* or *GAPDH* using the following formula: S/N ratio = %Input of positive region (*GLuc* or *SETD7*)/%Input of negative region (*CLuc* or *GAPDH*). The experiment was repeated three times independently.

**Library preparation**

2 μl of 14 μl eluted RNA was reverse transcribed with High-Capacity cDNA Reverse Transcription Kit (Thermo Fisher Scientific). IP efficiency was assessed by *GLuc*/*CLuc* or *SETD7*/*GAPDH* real time PCR. Once successfully immunoprecipitated methylated RNA could be confirmed, further transcriptome wide interrogation was pursued by deep sequencing using SMARTer Stranded Total RNA-Seq Kit v2 - Pico Input Mammalian (634413, Takara – Clontech, Japan) according to the manufacturer's protocol. Briefly, 3.5 μl of 14 μl eluted RNA and 50 ng input RNA were used for library construction, entering the protocol without fragmentation by adding first-strand cDNA synthesis mix. From that point on, the exact steps of the SMARTer Stranded Total RNA-Seq Kit v2 - Pico Input Mammalian User Manual were followed to the end. Libraries for IP RNA were PCR amplified for 16 cycles whereas 12 cycles was used for input RNA (the optimal cycle number need to be determined by the users). A purified library was quantified using a Qubit Fluorometer (Thermo Fisher Scientific), and the size distribution was checked using TapeStation D1000 ScreenTape (Agilent Technologies). The samples were then sequenced using a NextSeq 500 High Output Mode 75 cycles kit (Illumina, San Diego, CA) as single ends. Adapter sequences were removed, and sequences were demultiplexed using the bcl2fastq v.2 software (Illumina).
